# AgOTf-catalyzed one-pot reactions of 2-alkynylbenzaldoximes with α,β-unsaturated carbonyl compounds

**DOI:** 10.3762/bjoc.9.231

**Published:** 2013-09-27

**Authors:** Qiuping Ding, Dan Wang, Puying Luo, Meiling Liu, Shouzhi Pu, Liyun Zhou

**Affiliations:** 1 Key Laboratory of Functional Small Organic Molecules, Ministry of Education and College of Chemistry & Chemical Engineering, Jiangxi Normal University, Nanchang, Jiangxi 330022, P. R. China; 2 Department of Obstetrics and Gynecology, Jiangxi Provincial people's Hospital, Nanchang, Jiangxi 330006, P. R. China; 3 Jiangxi Key Laboratory of Organic Chemistry, Jiangxi Science & Technology Normal University, Nanchang, Jiangxi 330013, P. R. China

**Keywords:** 2-alkynylbenzaldoxime, cyclization, 2-(isoquinolin-1-yl)ethanol, rearrangement, α,β-unsaturated carbonyl compound

## Abstract

AgOTf-catalyzed one-pot reactions of 2-alkynylbenzaldoximes with various α,β-unsaturated carbonyl compounds under mild conditions are described, which provides a facile and efficient pathway for the synthesis of 1-alkylated isoquinoline derivatives. The method tolerates a wide range of substrates and allows for the preparation of the products of interest in moderate to excellent yields.

## Introduction

One-pot combinations of multi-catalysis and multi-component cascade reactions [1–6], in which several bond-forming steps take place in a single operation, play an important role in atom-economical organic chemistry. A cascade reaction is the most efficient way for targeting fine chemicals, agrochemicals, pharmaceutical drugs, drug intermediates and ingredients by a one-pot reaction in environmentally and economically friendly synthetic processes. Isoquinoline derivatives, an important class of nitrogen-containing polycyclic heteroarenes, have attracted considerable attention because of their pharmacological activities, including antitumor, antifungal, antimalarial, antihypertensive and antihistaminic activity, and their photo- and electrochemical properties [7–15]. Over the past decade, there has been growing interest in the development of new methods for the construction of isoquinoline. For instance, Yamamoto [16–19], Larock [20–27], and Wu [28–35] have reported mild and efficient methodologies to synthesize substituted isoquinolines.

Despite the aforementioned versatile and efficient methods for the direct construction of isoquinolines, the selective functionalization of isoquinoline species is still a challenging task. Recently, there has been some progress in this aspect. Wu and co-workers described an efficient three-component reaction of a 2-alkynylbenzaldoxime and an α,β-unsaturated carbonyl compound with bromine or iodine monochloride under mild conditions, which generates the 1-alkylated isoquinolines in good to excellent yields [36]. Wu and co-workers also reported many other highly functionalized isoquinoline derivatives by cascade reactions in good yields under mild conditions, such as 1-aminoisoquinolines [37] and 1-(isoquinolin-1-yl)ureas [38–39]. Recently, Deng and co-workers also described a new Pd-catalyzed C–H oxidation system for the regioselective alkylation of isoquinoline *N*-oxide and its derivatives with sulfoxides for the synthesis of 1-alkylated isoquinolines [40].

We also reported the synthesis of 1-arylated 1,3-disubstituted isoquinoline *N*-oxides in a one-pot reaction characterized by a Ag-catalyzed intramolecular addition cyclization/Pd-catalyzed direct arylation of 2-alkynylbenzaldoximes [41]. Inspired by the key contributions from the groups of Wu [36–39] and Deng [40], we envisioned that 1-alkylated isoquinolines could be generated in a one-pot AgOTf-catalyzed cyclization/1,3-dipolar cycloaddition/rearrangement or fragmentation from 2-alkynylbenzaldoximes and α,β-unsaturated carbonyl compounds.

**Scheme 1:**
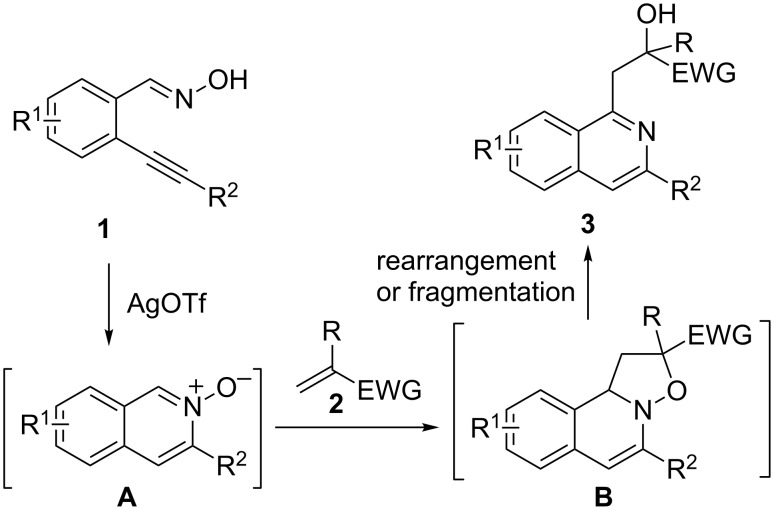
Proposed route for the AgOTf-catalyzed one-pot reaction of 2-alkynylbenzaldoxime with an α,β-unsaturated carbonyl compound.

Based on previous results [36–39,41–43], we expect 2-alkynylbenzaldoxime **1** to easily convert at room temperature to isoquinoline *N*-oxide **A** by a AgOTf-catalyzed cyclization. Compound **A** produced in situ might undergo a 1,3-dipolar cycloaddition with α,β-unsaturated carbonyl compound **2** leading to 2,10b-dihydro-1*H*-isoxazolo[3,2-*a*]isoquinoline intermediate **B** [44–45], which may then suffer a rearrangement or fragmentation resulting in compound **3** (Scheme 1) [35–36,38,42]. To demonstrate the feasibility of this assumed route, we started to investigate the possibility of this one-pot process.

## Results and Discussion

**Scheme 2:**
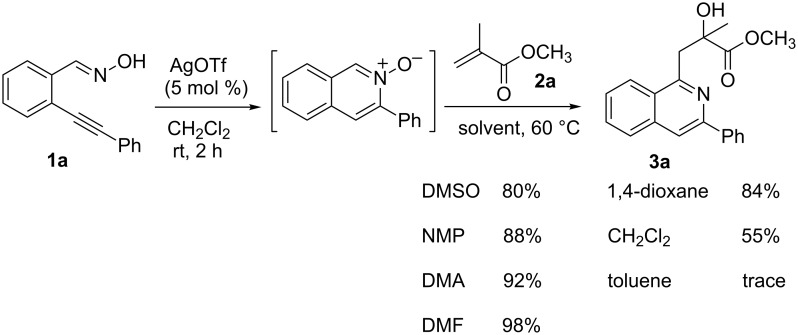
Synthesis of 1-alkylated isoquinoline **3a** by a AgOTf-catalyzed one-pot reaction in different solvents.

Initially, a set of experiments was carried out with 2-alkynylbenzaldoxime **1a** and methyl methacrylate (**2a**) as model substrates in the presence of AgOTf (5 mol %). As expected, the reaction proceeded smoothly in CH_2_Cl_2_ at room temperature to afford the desired product **3a** in 55% yield. We also tested other solvents, such as 1,4-dioxane, NMP, DMSO, DMA, toluene and DMF (Scheme 2). The solvent screening demonstrated that DMF was the best choice for the reaction at 60 °C. From these results, it was found that this one-pot process was highly efficient to construct 1-alkylated isoquinolines under very mild conditions.

With the optimized conditions in hand, the scope of the procedure was investigated for the reaction of 2-(*p*-tolylethynyl)benzaldehyde oxime (**1b**) with a series of α,β-unsaturated carbonyl compounds **2a–g** (Table 1). In most cases, substrate **1b** reacted with α,β-unsaturated carbonyl compounds **2** leading to the corresponding 1-alkylated isoquinolines in moderate to excellent yields. For instance, the reaction with **2b** under standard conditions gave rise to the desired product **3c** in 81% yield (Table 1, entry 2). An excellent yield was observed when butyl acrylate (**2e**) was utilized in the reaction (98% yield, Table 1, entry 5). When *tert-*butyl acrylate (**2f**) was employed, the reaction led to the formation of the desired 1-alkylated product **3g** (80% yield, Table 1, entry 6) with a molar ratio of *syn-* and *anti*-isomers of ~1/6. It is noteworthy, that in other cases only a single product was observed. But-3-en-2-one (**2g**) was less reactive than the investigated acrylic acid esters and delivered the desired product only in moderate yield (40%, Table 1, entry 7). On the other hand, when substrate **1b** was treated with acrylonitrile **2h** under such conditions, the starting materials were recovered almost completely (Table 1, entry 8).

**Table 1:** AgOTf-catalyzed one-pot reactions of 2-(*p*-tolylethynyl)benzaldehyde oxime (**1b**) with α,β-unsaturated carbonyl compounds **2**.

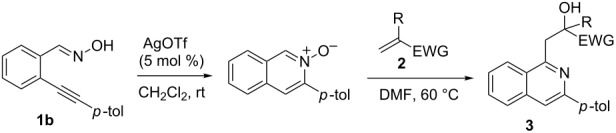			

Entry	**2**	Product **3**	Yield^a^ (%)

1	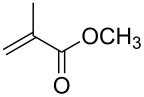 **2a**	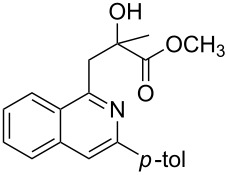 **3b**	70
2	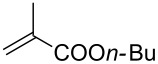 **2b**	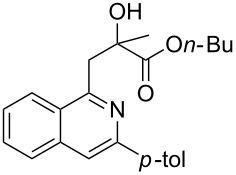 **3c**	81
3	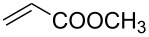 **2c**	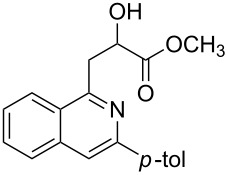 **3d**	50
4	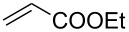 **2d**	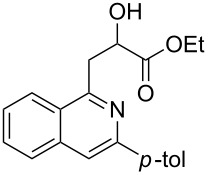 **3e**	61
5	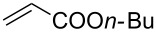 **2e**	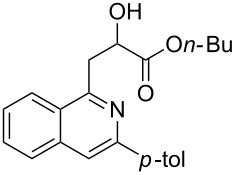 **3f**	98
6	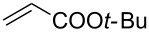 **2f**	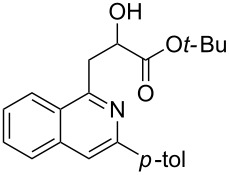 **3g**	80 (1/6)^b^
7	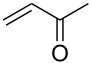 **2g**	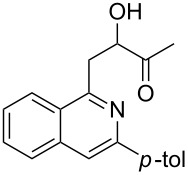 **3h**	40
8	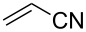 **2h**	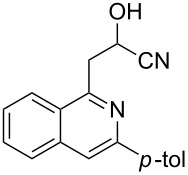 **3i**	–

^a^Isolated yield based on **1b**. ^b^Ratio of *syn*/*anti*, determined by ^1^H NMR.

Next, we examined the effect of substituents at the 2-alkynylbenzaldoxime **1**. In most cases, 2-alkynylbenzaldoxime **1** reacted with acrylates **2** leading to the desired products **3** in moderate to good yields. For instance, reaction of 2-((4-methoxyphenyl)ethynyl)benzaldehyde oxime (**1c**) with methyl methacrylate (**2a**) under the conditions described above gave the desired product **3j** in 75% yield (Table 2, entry 1). A better yield was obtained when substrate **1e** was employed in the reaction (83% yield, Table 2, entry 3). The usage of 2-(cyclopropylethynyl)benzaldehyde oxime (**1f**) in the reaction led to a similar yield (80% yield, Table 2, entry 5). However, low yields were obtained when 2-(hex-1-yn-1-yl)benzaldehyde oxime (**1g**) reacted with methyl methacrylate (**2a**). In the case of substrate **1h** (R^2^ = SiMe_3_) only desilyl product **3p** was observed in poor yield due to the instability of the product. When R^2^ was changed to H (2-ethynylbenzaldehyde oxime (**1i**), Table 2, entry 8), there was no reaction at all. Good yields were obtained when 2-alkynylbenzaldoximes substituted with other electron-withdrawing groups (such as **1j** and **1k**) reacted with acrylate **2a** (Table 2, entries 9–12). However, substrates with electron-donating groups attached on the aromatic ring of 2-alkynylbenzaldoxime (such as substrate **1l**, Table 2, entry 13) did not afford a desired product.

**Table 2:** One-pot reactions of 2-alkynylbenzaldoximes **1** with acrylates **2**.

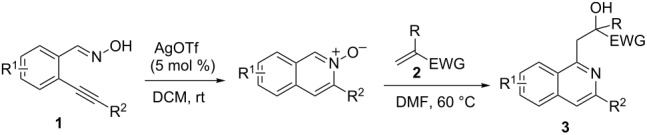				

Entry	2-Alkynylbenzaldoxime **1**	**2**	Product **3**	Yield^a^ (%)

1	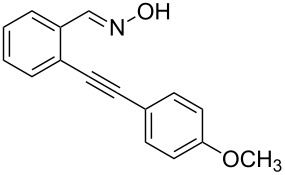 **1c**	**2a**	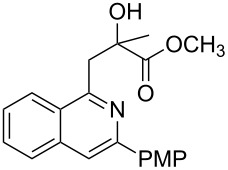 **3j**	75
2	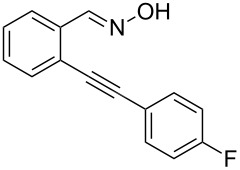 **1d**	**2a**	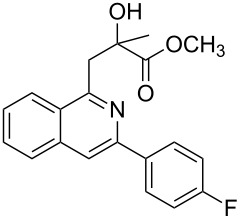 **3k**	48
3	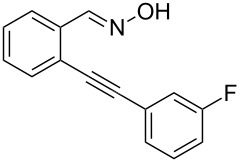 **1e**	**2a**	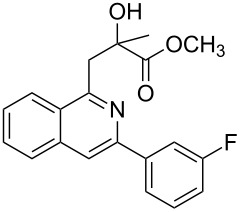 **3l**	83
4	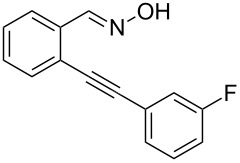 **1e**	**2f**	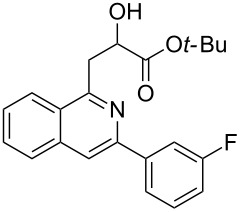 **3m**	70 (1/4)^b^
5	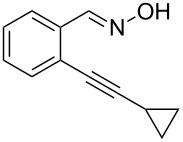 **1f**	**2a**	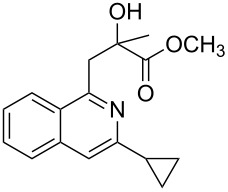 **3n**	80
6	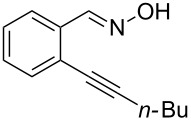 **1g**	**2a**	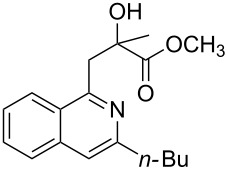 **3o**	35
7	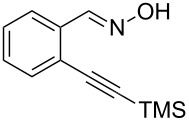 **1h**	**2a**	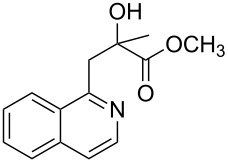 **3p**	12
8	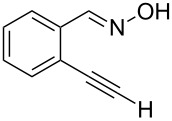 **1i**	**2a**	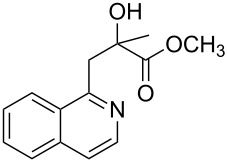 **3p**	–
9	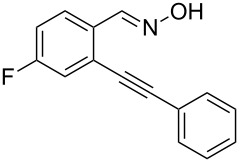 **1j**	**2a**	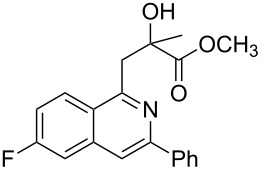 **3q**	80
10	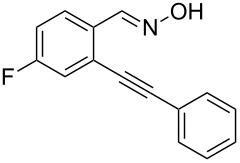 **1j**	**2e**	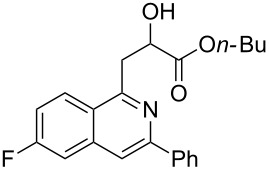 **3r**	72
11	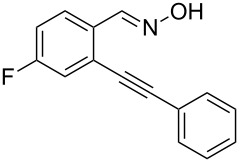 **1j**	**2f**	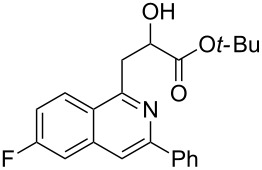 **3s**	71
12	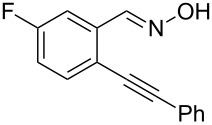 **1k**	**2a**	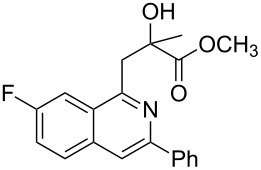 **3t**	85
13	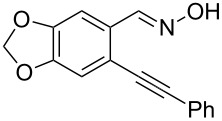 **1l**	**2a**	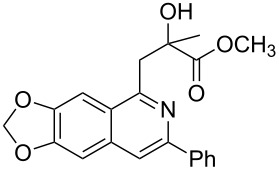 **3u**	–

^a^Isolated yield based on **1b**. ^b^Ratio of *syn*/*anti*, determined by ^1^H NMR.

**Scheme 3:**
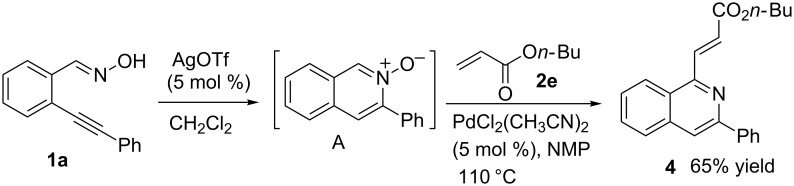
Pd-catalyzed one-pot alkenylation reaction of 2-alkynylbenzaldoxime **1a** and butyl acrylate (**2e**).

Recently, 1-alkenylated isoquinoline **4** was synthesized via a Pd-mediated C–H bond activation approach in a one-pot reaction. The intermediate isoquinoline *N*-oxide **A** was produced in situ from 2-alkynylbenzaldoximes and reacted with the α,β-unsaturated carbonyl compound **2e** to yield 1-alkenylated isoquinoline **4** (Scheme 3). This observation indicated that the Palladium-catalyzed alkenylation reaction mechanism might be similar to that described by Cui and Wu [46].

## Conclusion

In summary, we have demonstrated that one-pot reactions of 2-alkynylbenzaldoximes with α,β-unsaturated carbonyl compounds catalyzed by AgOTf occur smoothly under mild conditions. The present method provides a facile and efficient pathway for the synthesis of 1-alkylated isoquinoline derivatives in moderate to excellent yields with a wide range of substrates. The present one-pot catalyst system was also found to be applicable to the synthesis of 1-alkenylated isoquinoline derivatives.

## Experimental

### General

All reactions were performed in test tubes under a nitrogen atmosphere. Flash column chromatography was performed with silica gel (200–300 mesh). Analytical thin-layer chromatography was performed on glass plates pre-coated with 0.25 mm 230–400 mesh silica gel and impregnated with a fluorescent indicator (254 nm). Spots on thin-layer chromatography plates were visualized by exposure to ultraviolet light. Organic solutions were concentrated on rotary evaporators at 25–35 °C. Commercial reagents and solvents were used as received. ^1^H and ^13^C NMR spectra were recorded on a Bruker AV 400 spectrometer at 400 MHz (^1^H) and 100 MHz (^13^C) at ambient temperature. Chemical shifts are reported in parts per million (ppm) on the delta scale (δ) and referenced to tetramethylsilane (0 ppm). HRMS analyses were performed in ESI mode on a Bruker mass spectrometer.

General procedure for the AgOTf-catalyzed one-pot reactions of 2-alkynylbenzaldoximes **1** with α,β-unsaturated carbonyl compounds **2**: A mixture of 2-alkynylbenzaldoximes **1** (0.3 mmol) and AgOTf (0.015 mmol, 5 mol %) in CH_2_Cl_2_ (2 mL) was stirred at room temperature for 2 h, until 2-alkynylbenzaldoxime **1** was completely consumed. The solvent was removed under reduced pressure. Then, α,β-unsaturated carbonyl compound **2** (1.5 mmol, 5.0 equiv) in DMF (1 mL) was added to the residue, and allowed to stir at 60 °C overnight under a nitrogen atmosphere. After completion of the reaction as indicated by TLC, the reaction was quenched by water and extracted with ethyl acetate. The organic layers were dried with anhydrous MgSO_4_, the solvent was evaporated under reduced pressure, and the residue was purified by column chromatography with EtOAc/petroleum ether (1:5, v/v) as an eluent to yield the desired products **3**.

Procedure for the synthesis of 1-alkenylated isoquinoline **4** by a Pd-mediated C–H bond activation approach: A solution of 2-alkynylbenzaldoxime **1a** (0.3 mmol) and AgOTf (0.015 mmol, 5 mol %) in CH_2_Cl_2_ (2 mL) was stirred at rt for 2 h. Then, the solvent was removed under reduced pressure. Subsequently, a solution of α,β-unsaturated carbonyl compound **2e** (1.5 mmol, 5.0 equiv) and PdCl_2_(PhCN)_2_ (5 mol %) in NMP (1 mL) was added to the residue, and allowed to stir overnight at 110 °C under a nitrogen atmosphere. After completion of the reaction as indicated by TLC, the reaction was quenched by water and extracted with ethyl acetate. The organic layers were dried with anhydrous MgSO_4_, the solvent was evaporated under reduced pressure. The residue was purified by column chromatography with EtOAc/petroleum ether (1:3, v/v) as an eluent to yield the desired products **4**. For details, see Supporting Information File 1.

## Supporting Information

File 1 Experimental part.
