# Supplementary material for: AgOTf-catalyzed one-pot reactions of 2-alkynylbenzaldoximes with α,β-unsaturated carbonyl compounds
Source: Beilstein J Org Chem. 2013 Sep 27;9:1949–56. doi: 10.3762/bjoc.9.231 (PMC3817556; doi:10.3762/bjoc.9.231)
Supplement: File 1 — Experimental part. [file Beilstein_J_Org_Chem-09-1949-s001.pdf]

Supporting Information

for

AgOTf-catalyzed one-pot reactions of 2-alkynylbenzaldoximes with α,β-unsaturated carbonyl compounds

Qiuping Ding[1], Dan Wang[1], Puying Luo*[2], Meiling Liu[1], Shouzhi Pu*[3] and Liyun Zhou[1]

Address: [1]Key Laboratory of Functional Small Organic Molecules, Ministry of Education and College of Chemistry & Chemical Engineering, Jiangxi Normal University, Nanchang, Jiangxi 330022, P. R. China, [2]Department of Obstetrics and Gynecology, Jiangxi Provincial people's Hospital, Nanchang, Jiangxi 330006, P. R. China and [3]Jiangxi Key Laboratory of Organic Chemistry, Jiangxi Science & Technology Normal University, Nanchang, Jiangxi 330013, P. R. China

Email: Puying Luo - luopuying1979@gmail.com; Shouzhi Pu - pushouzhi@tsinghua.org.cn

*Corresponding author

Experimental part

1. General procedure and characterization data for compounds **3** and **4** S2–S13

2. NMR spectra of all compounds S14–S33

General procedure for the AgOTf-catalyzed one-pot reactions of 2-alkynylbenzaldoximes **1** with α,β-unsaturated carbonyl compounds **2**: A mixture of 2-alkynylbenzaldoximes **1** (0.3 mmol) and AgOTf (0.015 mmol, 5 mol %) in CH_2Cl_2 (2 mL) was stirred at room temperature for 2 h, until 2-alkynylbenzaldoxime **1** was completely consumed. The solvent was removed under reduced pressure. Then, α,β-unsaturated carbonyl compound **2** (1.5 mmol, 5.0 equiv) in DMF (1 mL) was added to the residue, and allowed to stir at 60 °C overnight under a nitrogen atmosphere. After completion of the reaction as indicated by TLC, the reaction was quenched by water and extracted with ethyl acetate. The organic layers were dried with anhydrous $MgSO_4$, the solvent was evaporated under vacuum, and the residue was isolated by column chromatography with EtOAc/petroleum ether (1/5, v/v) as eluent to yield the desired products **3**.

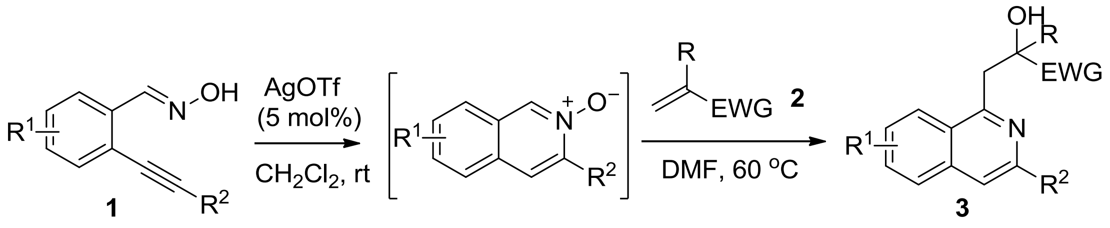

General procedure for palladium-catalyzed alkenylation reaction (Scheme 3):

A mixture of 2-alkynylbenzaldoximes **1a** (0.3 mmol) and AgOTf (0.015 mmol, 5 mol %) in CH_2Cl_2 (2 mL) was stirred at room temperature for 2 h, until 2-alkynylbenzaldoxime **1a** was completely consumed. The solvent was removed under reduced pressure. Then, butyl acrylate (**2e**, 192 mg, 1.5 mmol, 5.0 equiv), and $PdCl_2(CH_3CN)_2$ (3.9 mg, 5 mol %) in NMP (1 mL) was added to the residue, and

allowed to stir at 110 °C overnight under a nitrogen atmosphere. After completion of the reaction as indicated by TLC, the reaction was quenched by water and extracted with ethyl acetate. The organic layers were dried with anhydrous $MgSO_4$, the solvent was evaporated under vacuum, and the residue was isolated by column chromatography with EtOAc/petroleum ether (1/5, v/v) as eluent to yield the desired products **4**.

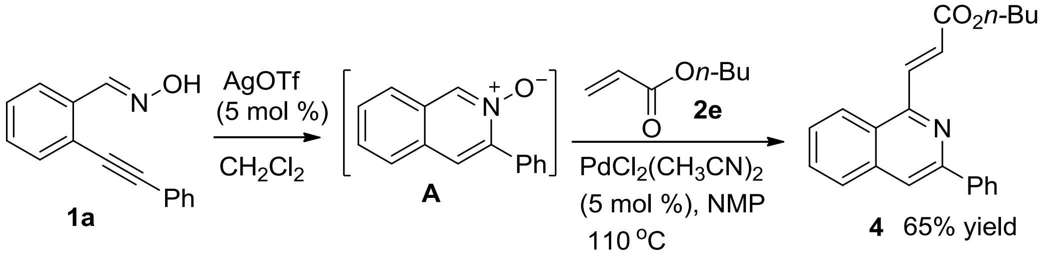

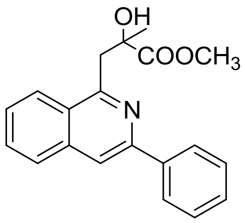

Methyl 2-hydroxy-2-methyl-3-(3-phenylisoquinolin-1-yl)propanoate **3a**: yield: 98%; white solid; R_f = 0.36; mp: 100–101 °C; ^{1}H NMR (400 MHz, $CDCl_3$) δ 1.69 (s, 3H), 3.48 (d, *J* = 16.8 Hz, 1H), 3.62 (s, 3H), 4.12 (d, *J* = 17.2 Hz, 1H), 6.53 (br, 1H), 7.38 (t, *J* = 7.2 Hz, 1H), 7.50-7.54 (m, 3H), 7.61 (t, *J* = 8.0 Hz, 1H), 7.77 (d, *J* = 8.0 Hz, 1H), 7.90 (s, 1H), 8.04-8.07 (m, 3H); ^{13}C NMR (100 MHz, $CDCl_3$) δ 26.8, 42.3, 52.3, 74.6, 115.7, 124.7, 126.3, 126.6, 127.4, 127.9, 128.8, 129.0, 130.7, 137.0, 138.7, 148.7, 158.3, 177.0; IR (KBr) υ /cm^{-1}: 3439, 2979, 2928, 1729, 1594, 1499; HRMS (ESI): *m/z* $[M + H]^+$ calcd for $C_{20}H_{20}NO_3$: 322.1443; found: 322.1449.

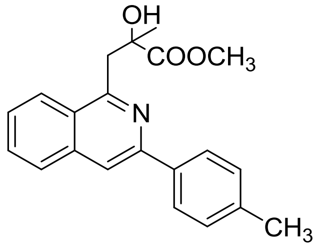

Methyl 2-hydroxy-2-methyl-3-(3-*p*-tolylisoquinolin-1-yl)propanoate (**3b**): yield: 70%; gray solid; R_f = 0.39; mp: 124–126 °C; ^{1}H NMR (400 MHz, $CDCl_3$) δ 1.70 (s, 3H), 2.40 (s, 3H), 3.49 (d, *J* = 16.8 Hz, 1H), 3.63 (s, 3H), 4.13 (d, *J* =17.2 Hz, 1H), 6.60 (br, 1H), 7.29 (d, *J* = 8.0 Hz, 2H), 7.54 (t, *J* = 7.2 Hz, 1H), 7.65 (t, *J* = 7.2 Hz, 1H), 7.81 (d, *J* = 8.0 Hz, 1H), 7.92 (s, 1H), 7.97 (d, *J* = 8.0 Hz, 2H), 8.08 (d, *J* = 8.4 Hz, 1H); ^{13}C NMR (100 MHz, $CDCl_3$) δ 21.3, 26.8, 42.3, 52.3, 74.6, 115.2, 124.7, 126.2, 126.5, 127.2, 127.9, 129.7, 130.7, 135.9, 137.1, 138.8, 148.8, 158.2, 177.0; IR (KBr) υ /cm^{-1}: 3461, 2975, 2951, 1747, 1590, 1495; HRMS (ESI): *m/z* $[M + H]^+$ calcd for $C_{21}H_{22}NO_3$: 336.1600; found: 336.1603.

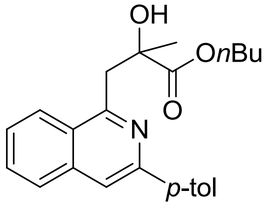

Butyl 2-hydroxy-2-methyl-3-(3-*p*-tolylisoquinolin-1-yl)propanoate (**3c**)**:** yield: 81%; brown solid; R_f = 0.50; mp: 89–90 °C; ^{1}H NMR (400 MHz, $CDCl_3$) δ 0.64 (t, *J* = 7.2 Hz, 3H), 0.99-1.05 (m, 2H), 1.29-1.40 (m, 2H), 1.71 (s, 3H), 2.40 (s, 3H), 3.48 (d, *J* = 16.8 Hz, 1H), 3.93-3.98 (m, 1H), 4.01-4.05 (m, 1H), 4.14 (d, *J* = 16.4 Hz, 1H), 6.64 (br, 1H), 7.30 (d, *J* = 8.0 Hz, 2H), 7.53 (t, *J* = 7.2 Hz, 1H), 7.63 (t, *J* = 8.0 Hz, 1H), 7.79 (d, *J* = 8.0 Hz, 1H), 7.90 (s, 1H), 7.98 (d, *J* = 8.0 Hz, 2H), 8.08 (d, *J* = 8.0 Hz, 1H); ^{13}C NMR (100 MHz, $CDCl_3$) δ 13.4, 18.8, 21.3, 26.8, 30.5, 42.1, 64.8, 74.6, 115.2, 124.7, 126.1, 126.6, 127.1, 127.8, 129.6, 130.5, 136.0, 137.1, 138.6, 148.9, 158.4, 176.4; IR (KBr) υ /cm^{-1}: 3379, 2957, 2930, 1716, 1621, 1591; HRMS (ESI): *m/z* $[M + H]^+$ calcd for $C_{24}H_{28}NO_3$: 378.2069; found: 378.2073.

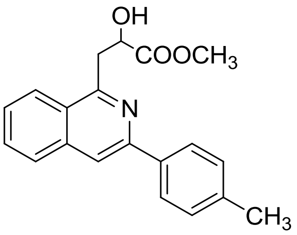

Methyl 2-hydroxy-3-(3-*p*-tolylisoquinolin-1-yl)propanoate (**3d**): yield: 50%; brown solid; R_f = 0.27; mp: 88–90 °C; ^{1}H NMR (400 MHz, $CDCl_3$) δ 2.41 (s, 3H), 3.75 (s, 3H), 3.85-3.87 (m, 2H), 4.95 (t, *J* = 4.8 Hz, 1H), 5.78 (br, 1H), 7.30 (d, *J* = 8.0 Hz, 2H), 7.56 (t, *J* = 7.6 Hz, 1H), 7.67 (t, *J* = 8.0 Hz, 1H), 7.83 (d, *J* = 8.0 Hz, 1H), 7.93 (s, 1H), 7.97 (d, *J* = 8.0 Hz, 2H), 8.07 (d, *J* = 8.4 Hz, 1H); ^{13}C NMR (100 MHz, $CDCl_3$) δ 21.3, 36.4, 52.3, 69.9, 115.4, 124.7, 126.2, 126.6, 127.2, 127.8, 129.7, 130.6, 136.1, 137.2, 138.7, 149.1, 157.9, 174.2; IR (KBr) υ /cm^{-1}: 3400, 2952, 2915, 1715, 1590, 1516; HRMS (ESI): *m/z* $[M + H]^+$ calcd for $C_{20}H_{20}NO_3$: 322.1443; found: 322.1437.

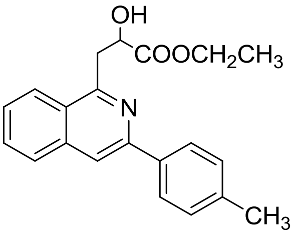

Ethyl 2-hydroxy-3-(3-*p*-tolylisoquinolin-1-yl)propanoate (**3e**): yield: 61%; brown solid; R_f = 0.33; mp: 102–103 °C; ^{1}H NMR (400 MHz, $CDCl_3$) δ 1.20 (t, *J* = 7.2 Hz, 3H), 2.43 (s, 3H), 3.84-3.90 (m, 2H), 4.21 (q, *J* = 7.2 Hz, 2H), 4.94 (t, *J* = 4.8 Hz, 1H), 5.78 (br, 1H), 7.31 (d, *J* = 8.0 Hz, 2H), 7.58 (t, *J* = 7.6 Hz, 1H), 7.69 (t, *J* = 8.0 Hz, 1H), 7.85 (d, *J* = 8.0 Hz, 1H), 7.95 (s, 1H), 7.99 (d, *J* = 8.0 Hz, 2H), 8.11 (d, *J* = 8.0 Hz, 1H); ^{13}C NMR (100 MHz, $CDCl_3$) δ 14.1, 21.3, 36.4, 61.3, 70.1, 115.4, 124.7, 126.2, 126.7, 127.2, 127.8, 129.6, 130.6, 136.1, 137.2, 138.7, 149.1, 157.9, 173.8; IR (KBr) υ /cm^{-1}: 3410, 2941, 2915, 1708, 1653, 1568; HRMS (ESI): *m/z* $[M + H]^+$ calcd for $C_{21}H_{22}NO_3$: 336.1600; found: 336.1596.

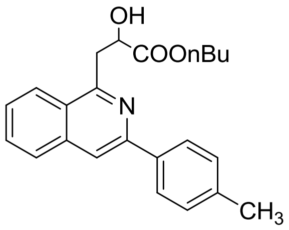

Butyl 2-hydroxy-3-(3-*p*-tolylisoquinolin-1-yl)propanoate (**3f**): yield: 98%; yellow solid; R_f = 0.34; mp: 66–67 °C; ^{1}H NMR (400 MHz, $CDCl_3$) δ 0.77 (t, *J* = 7.2 Hz, 3H), 1.16-1.22 (m, 2H), 1.47-1.53 (m, 2H), 2.42 (s, 3H), 3.82-3.92 (m, 2H), 4.10-4.15 (m, 2H), 4.93 (t, *J* = 4.8 Hz, 1H), 5.83 (br, 1H), 7.31 (d, *J* = 8.0 Hz, 2H), 7.57 (t, *J* = 7.2 Hz, 1H), 7.68 (t, *J* = 7.2 Hz, 1H), 7.85 (d, *J* = 8.4 Hz, 1H), 7.94 (s, 1H), 7.98 (d, *J* = 8.0 Hz, 2H), 8.10 (d, *J* = 8.0 Hz, 1H); ^{13}C NMR (100 MHz, $CDCl_3$) δ 13.6, 19.0, 21.3, 30.6, 36.5, 65.1, 70.1, 115.4, 124.8, 126.2, 126.7, 127.2, 127.9, 129.6, 130.6, 137.2, 138.7, 149.2, 158.0, 173.9; IR (KBr) υ /cm^{-1}: 3406, 2959, 2927, 1716, 1591, 1517; HRMS (ESI): *m/z* $[M + H]^+$ calcd for $C_{23}H_{26}NO_3$: 364.1913; found: 364.1918.

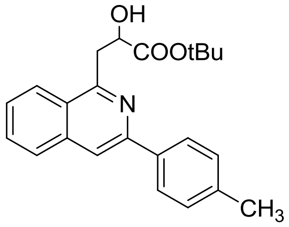

tert-Butyl 2-hydroxy-3-(3-*p*-tolylisoquinolin-1-yl)propanoate (**3g**): yield: 80%; yellow oil; R_f = 0.38; ^{1}H NMR (400 MHz, $CDCl_3$) δ 1.31 (s, 9H), 2.41 (s, 3H), 3.75 (dd, *J* = 4.0, 16.8 Hz, 1H), 3.88 (dd, *J* = 5.6, 16.8 Hz, 1H), 4.81 (t, *J* = 4.8 Hz, 1H), 5.58 (br, 1H), 7.30 (d, *J* = 8.0 Hz, 2H), 7.55 (t, *J* = 7.2 Hz, 1H), 7.66 (t, *J* = 8.0 Hz, 1H), 7.83 (d, *J* = 8.0 Hz, 1H), 7.92 (s, 1H), 8.00 (d, *J* = 8.0 Hz, 2H), 8.09 (d, *J* = 8.0 Hz, 1H); ^{13}C NMR (100 MHz, $CDCl_3$) δ 21.3, 27.8, 36.7, 70.4, 81.5, 115.2, 124.8, 126.2, 126.7, 127.1, 127.8, 129.6, 130.5, 136.1, 137.1, 138.6, 149.2, 158.2, 173.0; IR (KBr) υ /cm^{-1}: 3445, 2976, 2924, 1731, 1623, 1570; HRMS (ESI): *m/z* $[M + H]^+$ calcd for $C_{23}H_{26}NO_3$: 364.1913; found: 364.1915.

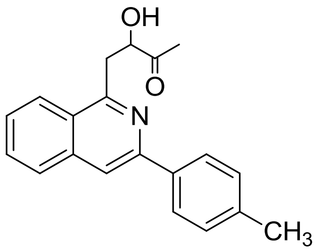

3-Hydroxy-4-(3-(*p*-tolyl)isoquinolin-1-yl)butan-2-one (**3h**): yield: 40%; yellow solid; R_f = 0.33; mp: 110–112 °C; ^{1}H NMR (400 MHz, $CDCl_3$) δ 2.41 (s, 3H), 2.42 (s, 3H), 3.71 (dd, *J* = 6.4, 16.4 Hz, 1H), 3.88 (dd, *J* = 3.2, 16.8 Hz, 1H), 4.73 (dd, *J* = 3.6, 6.4 Hz, 1H), 5.88 (br, 1H), 7.31 (t, *J* = 7.6 Hz, 2H), 7.56 (t, *J* = 7.6 Hz, 2H), 7.67 (t, *J* = 7.6 Hz, 1H), 7.83 (t, *J* = 8.4 Hz, 1H), 7.88 (d, *J* = 7.6 Hz, 1H), 7.91 (s, 1H), 8.12 (d, *J* = 8.0 Hz, 1H); ^{13}C NMR (100 MHz, $CDCl_3$) δ 21.2, 26.4, 35.7, 76.4, 115.7, 124.9, 126.3, 126.7, 127.2, 127.8, 129.6, 130.6, 136.2, 137.1, 138.7, 149.2, 158.2, 211.4; IR (KBr) υ /cm^{-1}: 3454, 3000, 2921, 1704, 1590, 1516; HRMS (ESI): *m/z* $[M + H]^+$ calcd for $C_{20}H_{20}NO_2$: 306.1494; found: 306.1498.

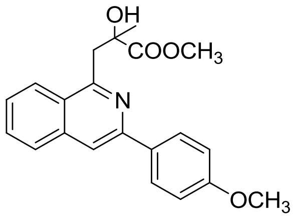

Methyl 2-hydroxy-3-(3-(4-methoxyphenyl)isoquinolin-1-yl)-2-methylpropanoate (**3j**): yield: 75%; yellow solid; R_f = 0.29; mp: 132–133 °C; ^{1}H NMR (400 MHz, $CDCl_3$) δ 1.69 (s, 3H), 3.49 (d, *J* = 16.8 Hz, 1H), 3.62 (s, 3H), 3.84 (s, 3H), 4.12 (d, *J* = 16.8 Hz, 1H), 6.57 (br, 1H), 7.01 (d, *J* = 8.8 Hz, 2H), 7.51 (t , *J* = 7.2 Hz, 3H), 7.62 (t, *J* = 7.2 Hz, 1H), 7.77 (d, *J* = 8.0 Hz, 1H), 7.84 (s, 1H), 8.02 (d, *J* = 8.8 Hz, 2H), 8.05 (d, *J* = 8.8 Hz, 1H); ^{13}C NMR (100 MHz, $CDCl_3$) δ 26.8, 42.3, 52.4, 55.4, 74.5, 114.3, 114.5, 124.7, 125.9, 127.0, 127.7, 127.9, 130.6, 131.3, 137.2, 148.5, 158.1, 160.2, 177.1; IR (KBr) υ /cm^{-1}: 3390, 2947, 2930, 1748, 1606, 1518; HRMS (ESI): *m/z* $[M + H]^+$ calcd for $C_{21}H_{22}NO_4$: 352.1549; found: 352.1552.

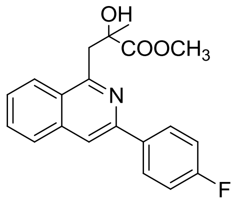

Methyl 3-(3-(4-fluorophenyl)isoquinolin-1-yl)-2-hydroxy-2-methylpropanoate (**3k**): yield: 48%; white solid; R_f = 0.31; mp: 126–127 °C; ^{1}H NMR (400 MHz, $CDCl_3$) δ 1.70 (s, 3H), 3.53 (d, *J* = 16.8 Hz, 1H), 3.62 (s, 3H), 4.12 (d, *J* = 16.8 Hz, 1H), 6.34 (br, 1H), 7.17 (t, *J* = 8.4 Hz, 2H), 7.58 (t, *J* = 8.0 Hz, 1H), 7.68 (t, *J* = 8.0 Hz, 1H), 7.83 (d, *J* = 8.4 Hz, 1H), 7.89 (s, 1H), 8.04 (dd, *J* = 5.6, 8.4 Hz, 2H), 8.09 (d, *J* = 8.4 Hz, 1H); ^{13}C NMR (100 MHz, $CDCl_3$) δ 26.8, 42.4, 52.3, 74.5, 115.4, 115.8 (d, $^2J_{C-F}$ = 22 Hz), 124.8, 126.2, 127.5, 127.8, 128.4 (d, $^2J_{C-F}$ = 8 Hz), 130.8, 134.9, 137.1, 147.8, 158.3, 163.3 (d, $^1J_{C-F}$ = 247 Hz), 176.9; IR (KBr) υ /cm^{-1}: 3373, 2986, 2979, 1727, 1601, 1587; HRMS (ESI): *m/z* $[M + H]^+$ calcd for $C_{20}H_{19}FNO_3$: 340.1349; found: 340.1353.

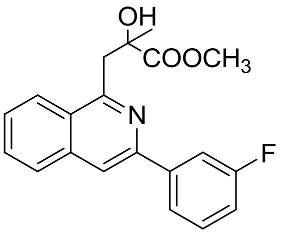

Methyl 3-(3-(3-fluorophenyl)isoquinolin-1-yl)-2-hydroxy-2-methylpropanoate (**3l**): yield: 83%; yellow solid; R_f = 0.36; mp: 127–129 °C; ^{1}H NMR (400 MHz, $CDCl_3$) δ 1.70 (s, 3H), 3.53 (d, *J* = 16.8 Hz, 1H), 3.67 (s, 3H), 4.13 (d, *J* = 16.8 Hz, 1H), 6.16 (br, 1H), 7.07 (dt, *J* = 2.0, 8.0 Hz, 1H), 7.41-7.46 (m, 1H), 7.57 (t , *J* = 7.6 Hz, 1H), 7.67 (t , *J* = 8.0 Hz, 1H), 7.76-7.79 (m, 1H), 7.82 (t, *J* = 8.0 Hz, 2H), 7.91 (s, 1H), 8.08 (d, *J* = 8.4 Hz, 1H); ^{13}C NMR (100 MHz, $CDCl_3$) δ 26.8, 42.6, 52.4, 74.5, 113.5 (d, $^2J_{C-F}$ = 23 Hz), 115.5 (d, $^2J_{C-F}$ = 21 Hz), 116.1, 122.1, 124.8, 126.5, 127.7, 127.9, 130.4 (d, $^3J_{C-F}$ = 8 Hz), 130.9, 136.9, 141.1 (d, $^3J_{C-F}$ = 7 Hz), 147.4, 158.4, 163.4 (d, $^1J_{C-F}$ = 244 Hz), 177.0; IR (KBr) υ /cm^{-1}: 3452, 2949, 2933, 1728, 1567, 1428; HRMS (ESI): *m/z* $[M + H]^+$ calcd for $C_{20}H_{19}FNO_3$: 340.1349; found: 340.1355.

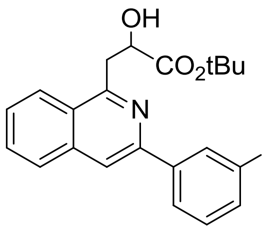

tert-Butyl 3-(3-(3-fluorophenyl)isoquinolin-1-yl)-2-hydroxypropanoate (**3m**): yield: 70%; brown solid; R_f = 0.34; mp: 96–98 °C; (*syn* + *anti*) ^{1}H NMR (400 MHz, $CDCl_3$) δ 1.32 (s, 7.2 H), 1.41 (s, 1.8 H), 3.78 (dd, *J* = 4.0, 16.4 Hz, 0.8 H), 3.88 (dd, *J* = 4.0, 16.4 Hz, 0.8 H), 4.30 (br, 0.2 H), 4.39 (dd, *J* = 4.8, 10.8 Hz, 0.2 H), 4.51 (dd, *J* = 4.8, 10.8 Hz, 0.2 H), 5.05 (br, 1H), 7.08 (dt, *J* = 1.2, 8.0 Hz, 1H), 7.42-7.47 (m, 1H), 7.60 (t , *J* = 7.6 Hz, 1H), 7.69 (t , *J* = 8.0 Hz, 1H), 7.79-7.90 (m, 3H), 7.94 (s, 0.8H), 7.98 (s, 0.2H), 8.07 (d, *J* = 8.8 Hz, 0.2H), 8.12 (d, *J* = 8.0 Hz, 0.8H); ^{13}C NMR (100 MHz, $CDCl_3$) (*anti*) δ 27.8, 37.1, 70.2, 81.8, 113.7 (d, $^2J_{C-F}$ = 23 Hz), 115.4 (d, $^2J_{C-F}$ = 21 Hz), 116.0, 122.4, 124.9, 126.7, 127.6, 127.9, 130.3 (d, $^3J_{C-F}$ = 8 Hz), 130.6, 136.9, 141.4 (d, $^3J_{C-F}$ = 8 Hz), 147.8, 158.3, 163.4 (d, $^1J_{C-F}$ = 243 Hz), 173.1; IR (KBr) υ /cm^{-1}: 3443, 2978, 2931, 1720, 1619, 1500; HRMS (ESI): *m/z* [M + H]$^+$ calcd for $C_{22}H_{23}FNO_3$: 368.1662; found: 368.1666.

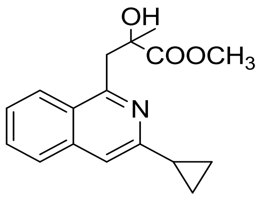

Methyl 3-(3-cyclopropylisoquinolin-1-yl)-2-hydroxy-2-methylpropanoate (**3n**): yield: 80%; brown oil; R_f = 0.34; ^{1}H NMR (400 MHz, $CDCl_3$) δ 0.99-1.05 (m, 4H), 1.62 (s, 3H), 2.08-2.15 (m, 1H), 3.37 (d, *J* = 16.8 Hz, 1H), 3.63 (s, 3H), 4.00 (d, *J* = 16.8 Hz, 1H), 6.50 (br, 1H), 7.40 (s, 1H), 7.48 (t, *J* = 7.6 Hz, 1H), 7.62 (t, *J* = 8.0 Hz, 1H), 7.69 (d, *J* = 8.0 Hz, 1H) , 8.02 (d, *J* = 8.8 Hz, 1H); ^{13}C NMR (100 MHz, $CDCl_3$) δ 9.0, 9.3, 16.7, 26.7, 41.6, 52.2, 74.6, 116.4, 124.7, 125.5, 126.2, 126.7, 130.4, 136.8, 153.7, 158.2, 176.8; IR (KBr) υ /cm^{-1}: 3397, 3002, 2951, 1747, 1592, 1498, 1404; HRMS (ESI): *m/z* [M + H]$^+$ calcd for $C_{17}H_{20}NO_3$: 286.1443; found: 286.1448.

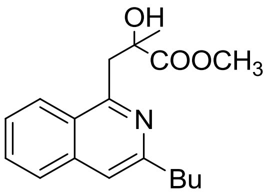

Methyl 3-(3-butylisoquinolin-1-yl)-2-hydroxy-2-methylpropanoate (**3o**): yield: 35%; brown oil; R_f = 0.50; ^{1}H NMR (400 MHz, $CDCl_3$) δ 0.95 (t, *J* = 7.6 Hz, 3H), 1.34-1.40 (m, 2H), 1.64 (s, 3H), 1.72-1.79 (m, 2H), 2.82-2.87 (m, 2H), 3.39 (d, *J* = 16.4 Hz, 1H), 3.61 (s, 3H), 4.05 (d, *J* = 16.4 Hz, 1H), 6.88 (br, 1H), 7.33 (s, 1H), 7.53 (t, *J* = 7.6 Hz, 1H), 7.63 (t, *J* = 7.2 Hz, 1H), 7.72 (d, *J* = 8.0 Hz, 1H), 8.06 (d, *J* = 8.4 Hz, 1H); ^{13}C NMR (100 MHz, $CDCl_3$) δ 13.9, 22.3, 26.7, 31.6, 37.3, 41.5, 52.1, 74.8, 117.4, 124.7, 125.6, 126.5, 127.0, 130.3, 137.0, 153.2, 158.1, 176.8; IR (KBr) υ /cm^{-1}: 3418, 2955, 2871, 1747, 1625, 1499; HRMS (ESI): *m/z* $[M + H]^+$ calcd for $C_{18}H_{24}NO_3$: 302.1756; found: 302.1763.

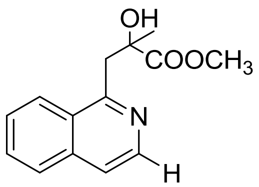

Methyl 2-hydroxy-2-methyl-3-(3-(trimethylsilyl)isoquinolin-1-yl)propanoate (**3p**): yield: 12%; brown solid; R_f = 0.26; mp: 69–71 °C; ^{1}H NMR (400 MHz, $CDCl_3$) δ 1.65 (s, 3H), 3.43 (d, *J* = 16.4 Hz, 1H), 3.60 (s, 1H), 4.06 (d, *J* = 16.4 Hz, 1H), 6.58 (br, 1H), 7.54 (d, *J* = 5.6 Hz, 1H), 7.63 (t, *J* = 7.6 Hz, 1H), 7.70 (t, *J* = 7.6 Hz, 1H), 7.82 (d, *J* = 8.4 Hz, 1H), 8.14 (d, *J* = 8.4 Hz, 1H), 8.34 (d, *J* = 6.0 Hz, 1H); ^{13}C NMR (100 MHz, $CDCl_3$) δ 26.8, 41.8, 52.3, 74.8, 114.1, 119.9, 124.9, 127.4, 127.5, 130.4, 136.2, 140.5, 158.6, 176.8; IR (KBr) υ /cm^{-1}: 3439, 2925, 2854, 1702, 1619, 1459; HRMS (ESI): *m/z* $[M + H]^+$ calcd for $C_{14}H_{16}NO_3$: 246.1130; found: 246.1127.

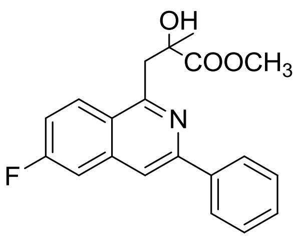

Methyl 3-(6-fluoro-3-phenylisoquinolin-1-yl)-2-hydroxy-2-methylpropanoate (**3q**): yield: 80%; yellow solid; R_f = 0.32; mp: 107–109 °C; ^{1}H NMR (400 MHz, $CDCl_3$) δ

1.69 (s, 3H), 3.49 (d, J = 16.8 Hz, 1H), 3.64 (s, 3H), 4.09 (d, J = 16.8 Hz, 1H), 6.35 (br, 1H), 7.31 (dt, J = 2.4, 8.8 Hz, 1H), 7.41 (t, J = 7.6 Hz, 2H), 7.49 (t, J = 7.6 Hz, 2H), 7.88 (s, 1H), 8.04 (d, J = 7.6 Hz, 1H), 8.12 (dd, J = 5.6, 8.8 Hz, 1H); ^{13}C NMR (100 MHz, $CDCl_3$) δ 26.3, 42.0, 51.9, 74.1, 110.5 (d, $^{2}J_{C\text{-}F}$ = 21 Hz), 114.7, 117.1 (d, $^{2}J_{C\text{-}F}$ = 25 Hz), 123.0, 126.2, 127.4 (d, $^{3}J_{C\text{-}F}$ = 9 Hz), 127.5, 128.4, 128.5, 137.8, 138.3 (d, $^{3}J_{C\text{-}F}$ = 10 Hz), 149.3, 157.7, 162.9 (d, $^{1}J_{C\text{-}F}$ = 252 Hz), 176.4; IR (KBr) ʋ /cm^{-1}: 3423, 2990, 2953, 1708, 1602, 1521; HRMS (ESI): m/z $[M + H]^+$ calcd for $C_{20}H_{19}FNO_3$: 340.1349; found: 340.1355.

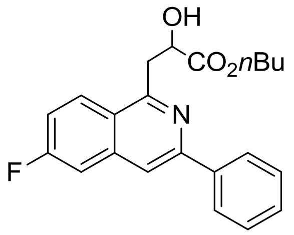

Butyl 3-(6-fluoro-3-phenylisoquinolin-1-yl)-2-hydroxypropanoate (**3r**): yield: 72%; yellow oil; R_f = 0.31; (*syn* + *anti*) ^{1}H NMR (400 MHz, $CDCl_3$) δ 0.78 (t, J = 7.2 Hz, 3H), 1.16-1.22 (m, 2H), 1.48-1.54 (m, 2H), 3.77-3.89 (m, 2H), 4.04-4.17 (m, 2H), 4.37-4.43 (m, 0.8H), 4.55 (dd, J = 4.4, 10.4 Hz, 0.4H), 4.71 (t, J = 4.0 Hz, 0.4H), 4.90 (t, J = 4.4 Hz, 0.6H), 5.44 (br, 0.6H), 7.31 (t, J = 8.4 Hz, 1H), 7.38-7.50 (m, 4H), 7.87 (s, 0.6H), 7.93 (s, 0.4H), 8.05 (d, J = 7.2 Hz, 2H), 8.10 (dd, J = 5.2, 9.2 Hz, 1H); ^{13}C NMR (100 MHz, $CDCl_3$) δ anti: 18.9, 30.5, 36.7, 63.3, 65.2, 69.9, 111.0 (d, $^{2}J_{C\text{-}F}$ = 20 Hz), 115.3 (d, $^{4}J_{C\text{-}F}$ = 4 Hz), 117.4, 117.7, 123.5, 126.9, 127.9 (d, $^{3}J_{C\text{-}F}$ = 10 Hz), 128.9, 138.5, 138.9 (d, $^{3}J_{C\text{-}F}$ = 10 Hz), 150.2, 158.0, 163.3 (d, $^{1}J_{C\text{-}F}$ = 251 Hz), 173.9; Syn: 18.9, 30.5, 51.2, 63.3, 65.2, 69.9, 111.1 (d, $^{2}J_{C\text{-}F}$ = 20 Hz), 115.9 (d, $^{4}J_{C\text{-}F}$ = 4 Hz), 117.7, 117.9, 123.6, 126.8, 127.6 (d, $^{3}J_{C\text{-}F}$ = 9 Hz), 129.0, 138.5, 139.1 (d, $^{3}J_{C\text{-}F}$ = 10 Hz), 150.3, 156.8, 163.2 (d, $^{1}J_{C\text{-}F}$ = 251 Hz), 171.2; IR (KBr) ʋ /cm^{-1}: 3454, 2959, 2932, 1738, 1503, 1455; HRMS (ESI): m/z $[M + H]^+$ calcd for $C_{22}H_{23}FNO_3$: 368.1662; found: 368.1667.

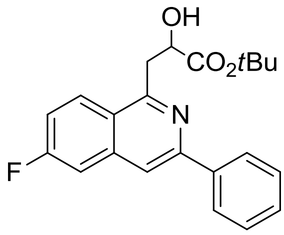

tert-Butyl 3-(6-fluoro-3-phenylisoquinolin-1-yl)-2-hydroxypropanoate (**3s**): yield: 71%; yellow oil; R_f = 0.34; (*syn* + *anti*) ^{1}H NMR (400 MHz, $CDCl_3$) δ 1.31 (s, 6.3H), 1.40 (s, 2.7H), 3.68 (s, 0.3H), 3.73 (dd, *J* = 4.0, 16.4 Hz, 0.7H), 3.85 (dd, *J* = 5.6, 16.4 Hz, 0.7H), 4.40 (dd, *J* = 4.8, 10.8 Hz, 0.3 H), 4.53 (dd, *J* = 4.8, 10.8 Hz, 0.3 H), 4.62 (t, *J* = 4.0 Hz, 0.3H), 4.80 (t, *J* = 4.4 Hz, 0.7H), 5.25 (br, 0.7H), 7.34 (t, *J* = 8.8 Hz, 1H), 7.39-7.52 (m, 4H), 7.89 (s, 0.7H), 7.94 (s, 0.3H), 8.06-8.15 (m, 3H); ^{13}C NMR (100 MHz, $CDCl_3$) δ 27.9, 37.1, 70.3, 81.9, 111.0 (d, $^2J_{C-F}$ = 20 Hz), 115.3 (d, $^4J_{C-F}$ = 5 Hz), 117.5 (d, $^2J_{C-F}$ = 21 Hz), 123.6, 127.0, 128.1 (d, $^3J_{C-F}$ = 10 Hz), 128.9, 138.6, 138.8 (d, $^3J_{C-F}$ = 10 Hz), 150.2, 158.2, 163.3 (d, $^1J_{C-F}$ = 251 Hz), 172.9; IR (KBr) υ /cm^{-1}: 3444, 2924, 2853, 1730, 1573, 1504; HRMS (ESI): *m/z* $[M + H]^+$ calcd for $C_{22}H_{23}FNO_3$: 368.1662; found: 368.1665.

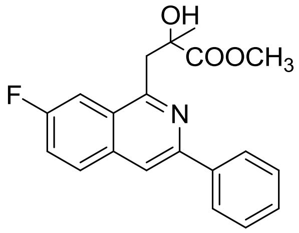

Methyl 3-(7-fluoro-3-phenylisoquinolin-1-yl)-2-hydroxy-2-methylpropanoate (**3t**): yield: 85%; yellow solid; R_f = 0.34; mp: 115–117 °C; ^{1}H NMR (400 MHz, $CDCl_3$) δ 1.70 (s, 3H), 3.44 (d, *J* = 16.8 Hz, 1H), 3.64 (s, 3H), 4.03 (d, *J* = 16.8 Hz, 1H), 6.30 (br, 1H), 7.37-7.50 (m, 4H), 7.67 (dd, *J* = 2.4, 9.6 Hz, 1H), 7.81 (dd, *J* = 5.6, 8.8 Hz, 1H), 7.91 (s, 1H), 8.03 (d, *J* = 7.2 Hz, 1H); ^{13}C NMR (100 MHz, $CDCl_3$) δ 26.8, 42.5, 52.4, 74.5, 108.5 (d, $^2J_{C-F}$ = 21 Hz), 115.3, 121.2 (d, $^2J_{C-F}$ = 24 Hz), 126.5, 126.8 (d, $^3J_{C-F}$ = 8 Hz), 126.9, 128.8, 128.9, 130.5 (d, $^3J_{C-F}$ = 9 Hz), 134.1, 138.4, 148.5, 157.6 (d, $^4J_{C-F}$ = 6 Hz), 160.9 (d, $^1J_{C-F}$ = 248 Hz), 176.9; IR (KBr) υ /cm^{-1}: 3406, 2998, 2958, 1747, 1595, 1507; HRMS (ESI): *m/z* $[M + H]^+$ calcd for $C_{20}H_{19}FNO_3$: 340.1394; found: 340.1399.

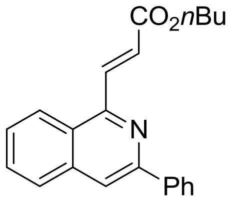

(*E*)-Butyl 3-(3-phenylisoquinolin-1-yl)-acrylate (**4**): yield: 65%; yellow solid; R_f = 0.75; mp: 58–59 °C; ^{1}H NMR (400 MHz, $CDCl_3$) δ 0.92 (t, *J* = 7.2 Hz, 3H), 1.35-1.42 (m, 2H),1.64-1.69 (m, 2H), 4.22 (t, *J* = 6.4 Hz, 2H), 7.36 (t, *J* = 6.0 Hz, 2H), 7.51 (t, *J* = 7.2 Hz, 2H), 7.55 (t, *J* = 7.2 Hz, 1H), 7.63 (t, *J* = 7.2 Hz, 1H), 7.83 (d, *J* = 8.4 Hz, 1H), 8.04 (s,1H), 8.15 (d, *J* = 7.6 Hz, 1H), 8.26 (d, *J* = 8.4 Hz, 1H), 8.50 (d, *J* = 15.2 Hz, 1H); ^{13}C NMR (100 MHz, $CDCl_3$) δ 13.8,19.2, 30.9, 64.8, 117.8, 124.1, 125.2, 126.5, 127.0, 127.6, 127.8, 128.8, 130.3, 130.9, 132.4, 137.7, 138.3, 139.1, 150.3, 151.4, 167.1; IR (KBr) υ/cm^{-1}: 3472, 2959, 2931, 1717, 1661, 1573; HRMS (ESI): *m/z* $[M + H]^+$ calcd for $C_{22}H_{22}NO_2$: 332.1651; found: 332.1657.

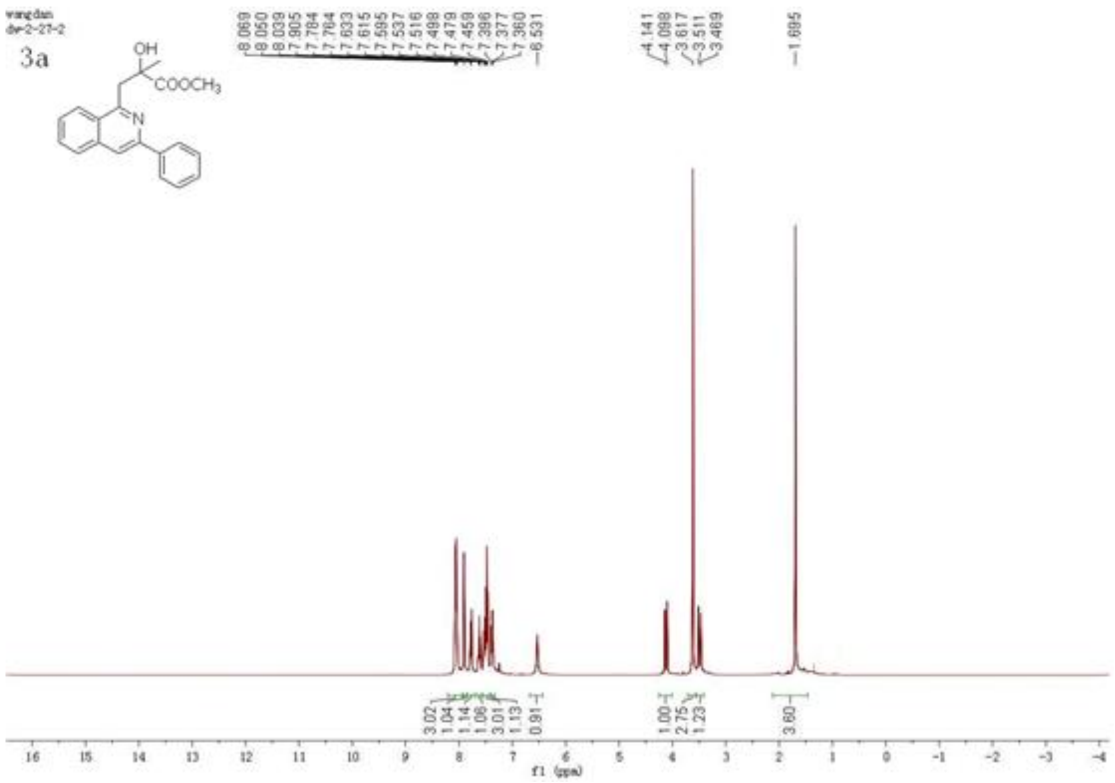
wangdan
dw-2-27-2
3a
OH
COOCH3
N
8.069
8.050
8.039
7.905
7.784
7.764
7.633
7.615
7.595
7.537
7.516
7.498
7.479
7.459
7.396
7.377
7.360
6.531
4.141
4.098
3.617
3.511
3.469
1.695
3.02
1.04
1.14
1.06
3.01
1.13
0.91
1.00
2.75
1.23
3.60
f1 (ppm)

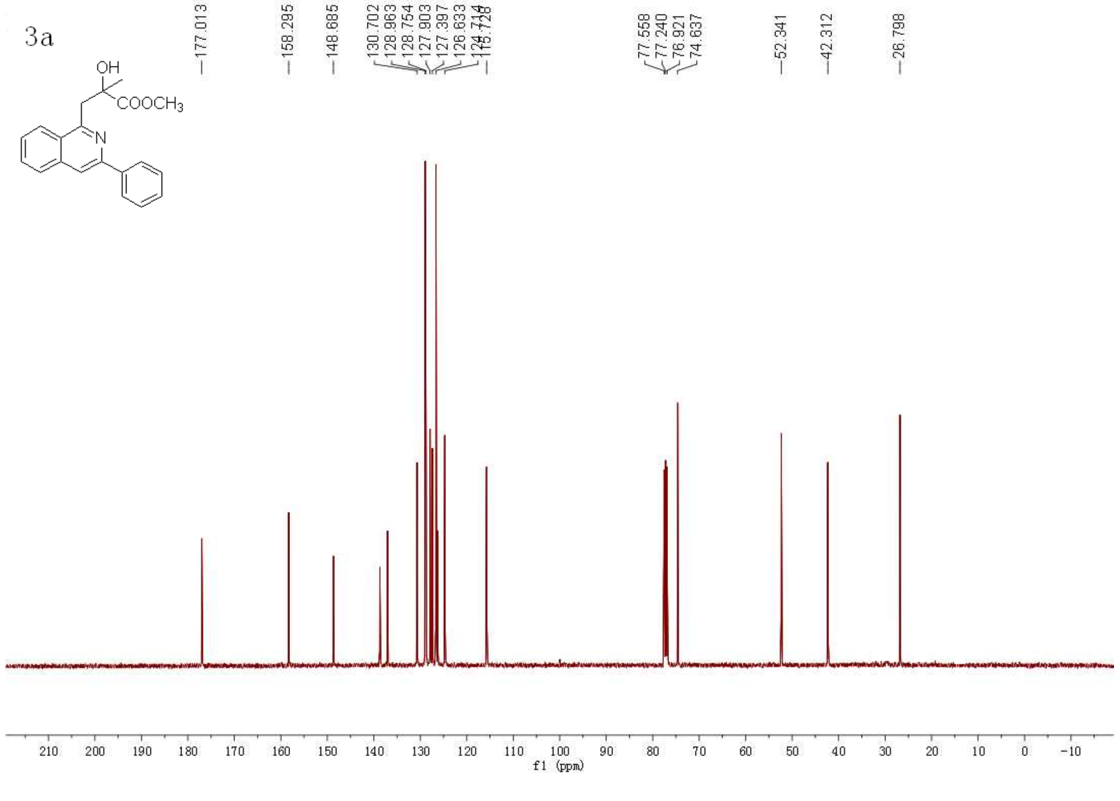
3a
OH
COOCH3
N
177.013
158.295
148.685
130.702
128.963
128.754
127.903
127.397
126.633
115.728
77.558
77.240
76.921
74.637
52.341
42.312
26.798
f1 (ppm)

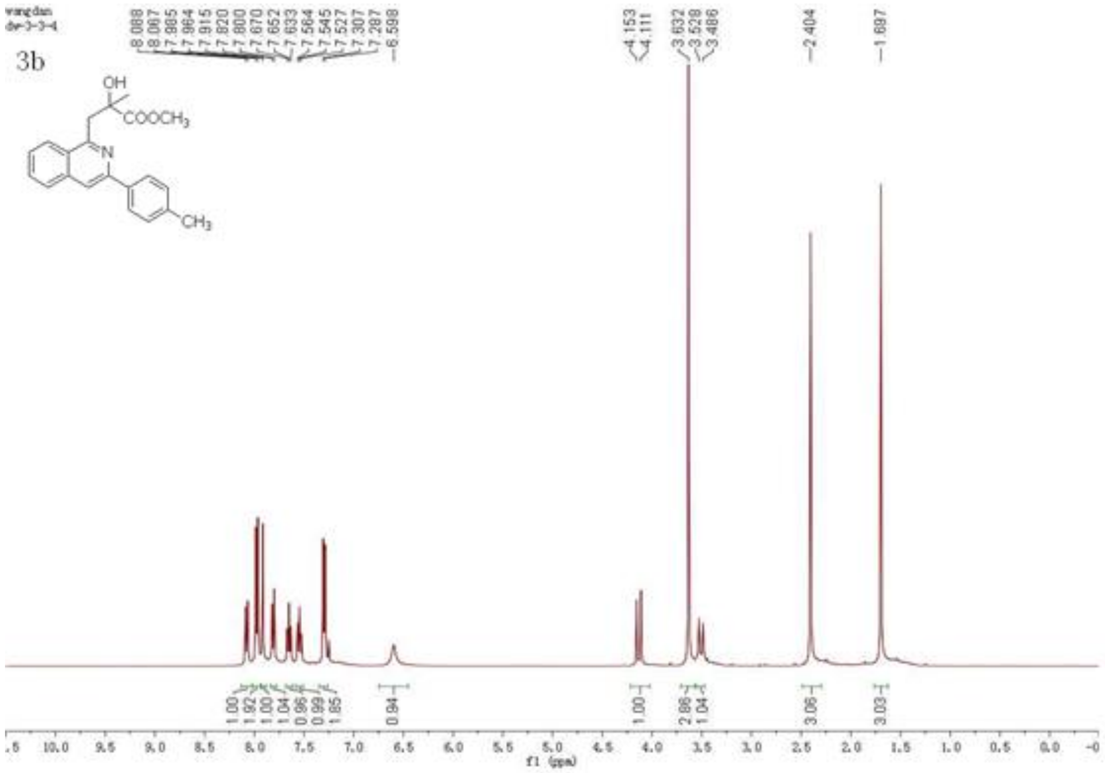
wangdan
dw-3-3-4
3b
OH
COOCH3
N
CH3
8.088
8.067
7.985
7.964
7.915
7.820
7.800
7.670
7.652
7.633
7.564
7.545
7.527
7.307
7.287
6.598
4.153
4.111
3.632
3.528
3.486
2.404
1.697
1.00
1.92
1.00
1.04
0.96
0.99
1.85
0.94
1.00
2.86
1.04
3.06
3.03
f1 (ppm)

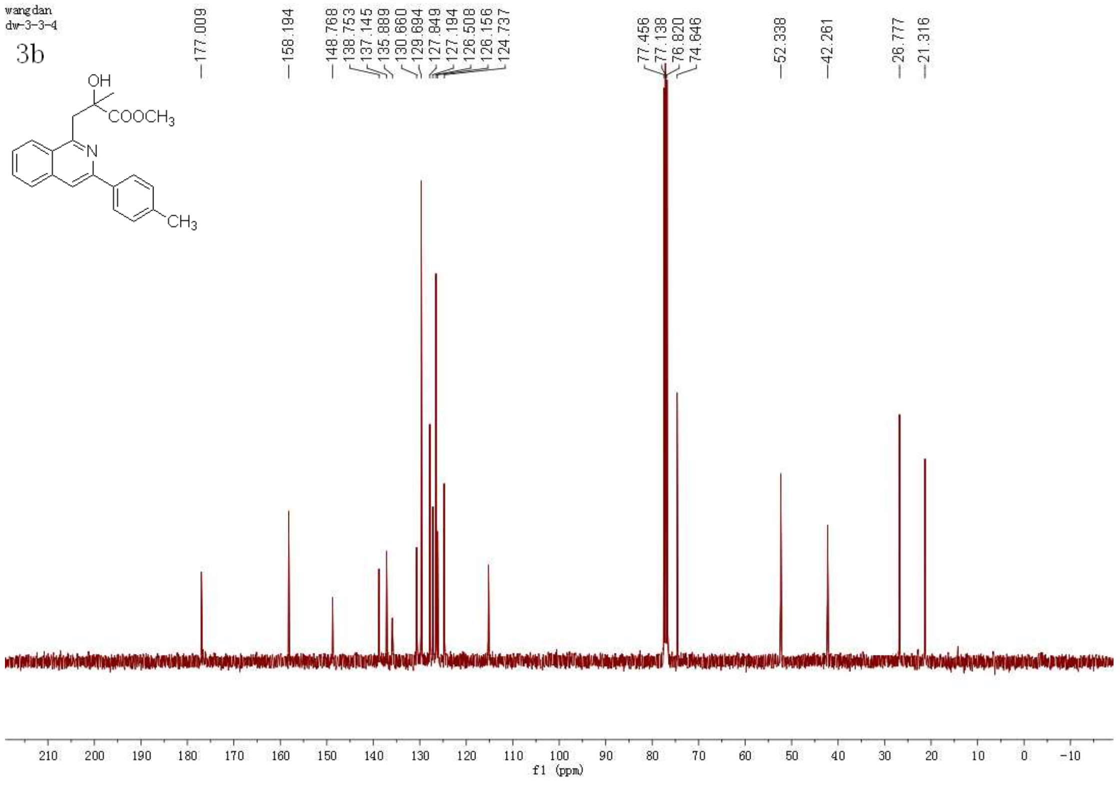
wangdan
dw-3-3-4
3b
OH
COOCH3
N
CH3
177.009
158.194
148.768
138.753
137.145
135.889
130.660
129.694
127.849
127.194
126.508
126.156
124.737
77.456
77.138
76.820
74.646
52.338
42.261
26.777
21.316
f1 (ppm)

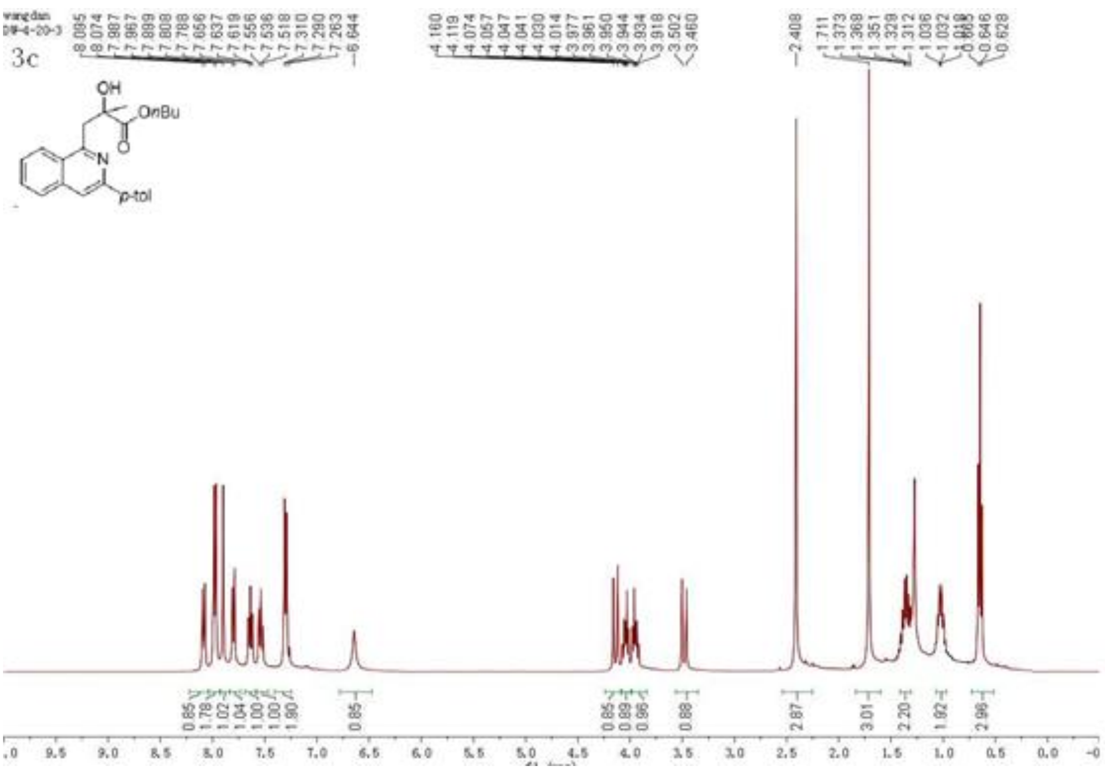
wangdan
DW-4-20-3
3c
OH
OnBu
O
N
p-tol
8.085 8.074 7.987 7.967 7.899 7.808 7.788 7.656 7.637 7.619 7.556 7.536 7.518 7.310 7.290 7.263 6.644
4.160 4.119 4.074 4.057 4.047 4.041 4.030 4.014 3.977 3.961 3.950 3.944 3.934 3.918 3.502 3.460
2.408 1.711 1.373 1.368 1.351 1.329 1.312 1.036 1.032 0.646 0.628
0.85 1.78 1.02 1.04 1.00 1.00 1.90 0.85 0.85 0.89 0.96 0.88 2.87 3.01 2.20 1.92 2.98

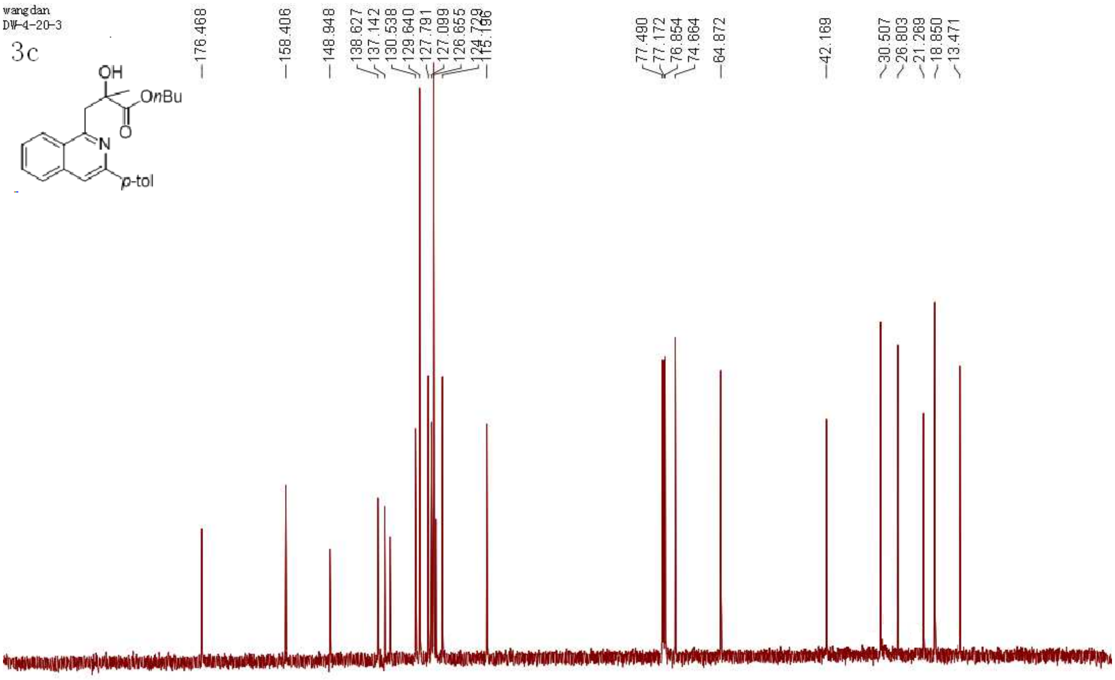
wangdan
DW-4-20-3
3c
OH
OnBu
O
N
p-tol
176.468 158.406 148.948 138.627 137.142 130.538 129.640 127.791 127.099 126.655 124.729 115.195
77.490 77.172 76.854 74.664 64.872 42.169 30.507 26.803 21.269 18.850 13.471
210 200 190 180 170 160 150 140 130 120 110 100 90 80 70 60 50 40 30 20 10 0 -10
f1 (ppm)

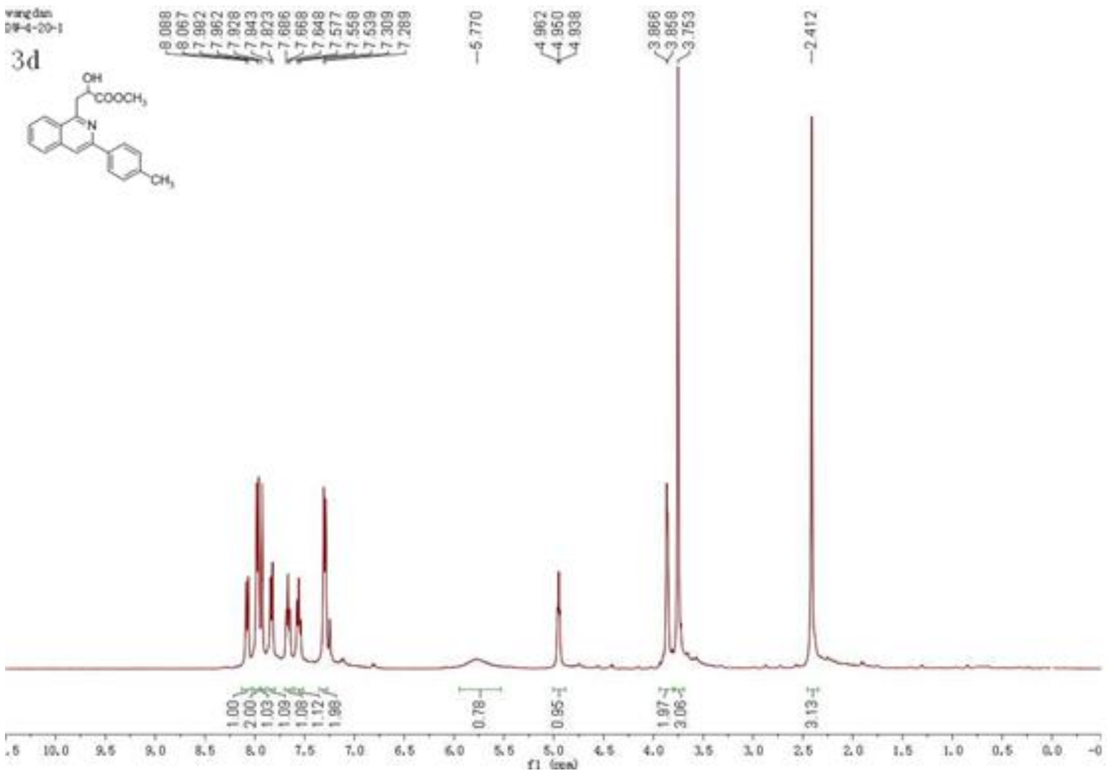
wangdan
DW-4-20-1
3d
OH
COOCH3
N
CH3
8.088
8.067
7.982
7.962
7.928
7.843
7.823
7.686
7.668
7.648
7.577
7.558
7.539
7.309
7.289
5.770
4.962
4.950
4.938
3.866
3.858
3.753
2.412
1.00
2.00
1.03
1.09
1.08
1.12
1.98
0.78
0.95
1.97
3.06
3.13
f1 (ppm)

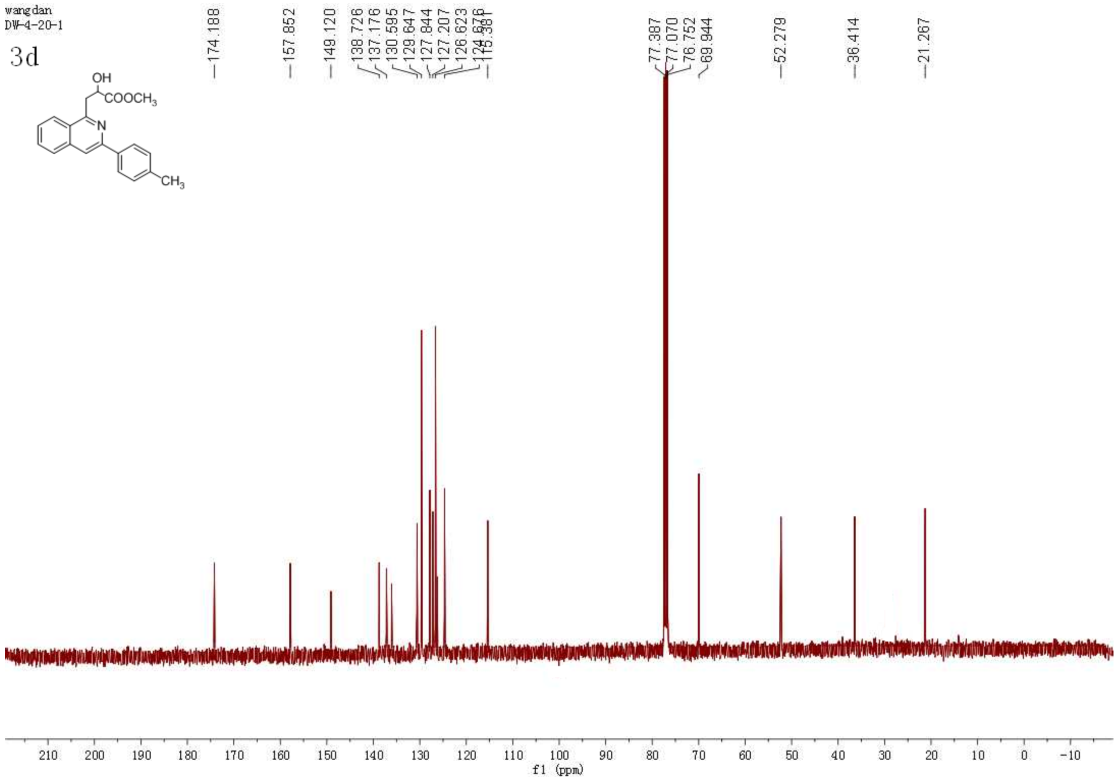
wangdan
DW-4-20-1
3d
OH
COOCH3
N
CH3
174.188
157.852
149.120
138.726
137.176
130.595
129.647
127.844
127.207
126.623
124.676
115.381
77.387
77.070
76.752
69.944
52.279
36.414
21.267
f1 (ppm)

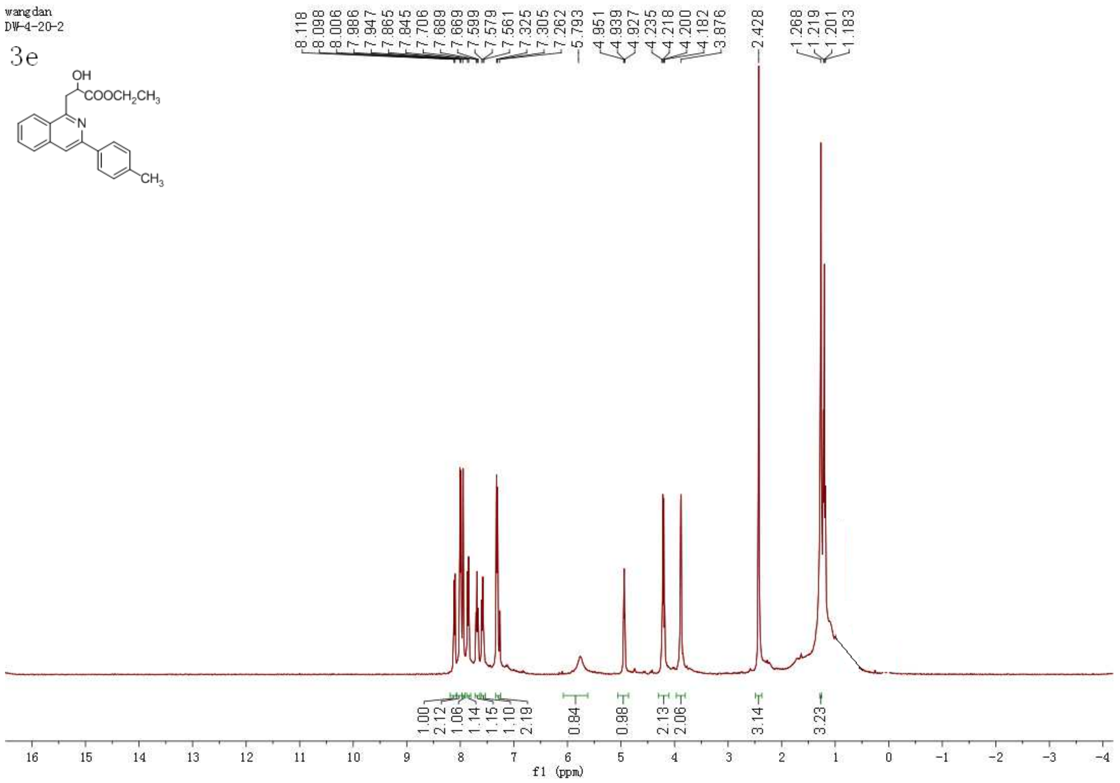

wangdan
DW-4-20-2
3e
OH
COOCH2CH3
N
CH3
8.118 8.098 8.006 7.986 7.947 7.865 7.845 7.706 7.689 7.669 7.599 7.579 7.561 7.325 7.305 7.262 5.793 4.951 4.939 4.927 4.235 4.218 4.200 4.182 3.876 2.428 1.268 1.219 1.201 1.183
1.00 2.12 1.06 1.14 1.15 1.10 2.19 0.84 0.98 2.13 2.06 3.14 3.23
f1 (ppm)

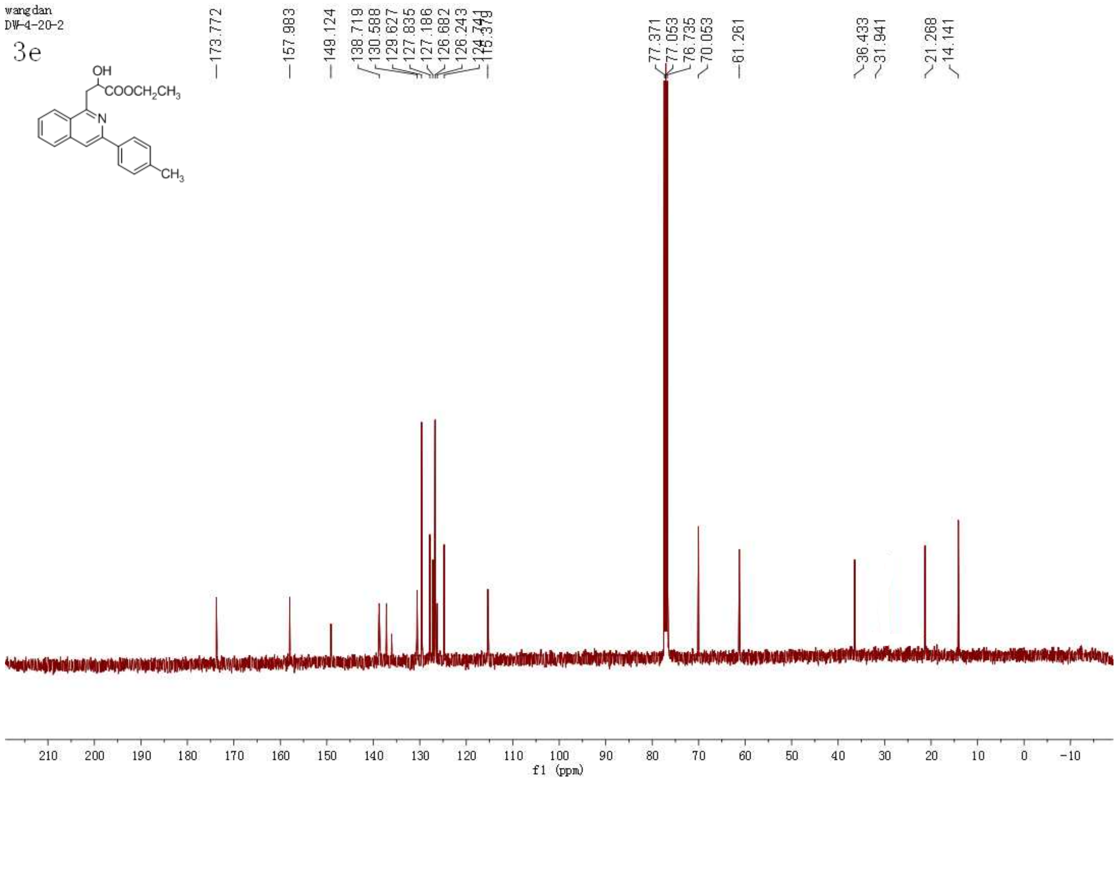

wangdan
DW-4-20-2
3e
OH
COOCH2CH3
N
CH3
173.772 157.983 149.124 138.719 130.588 129.627 127.835 127.186 126.682 126.243 124.741 115.379 77.371 77.053 76.735 70.053 61.261 36.433 31.941 21.268 14.141
f1 (ppm)

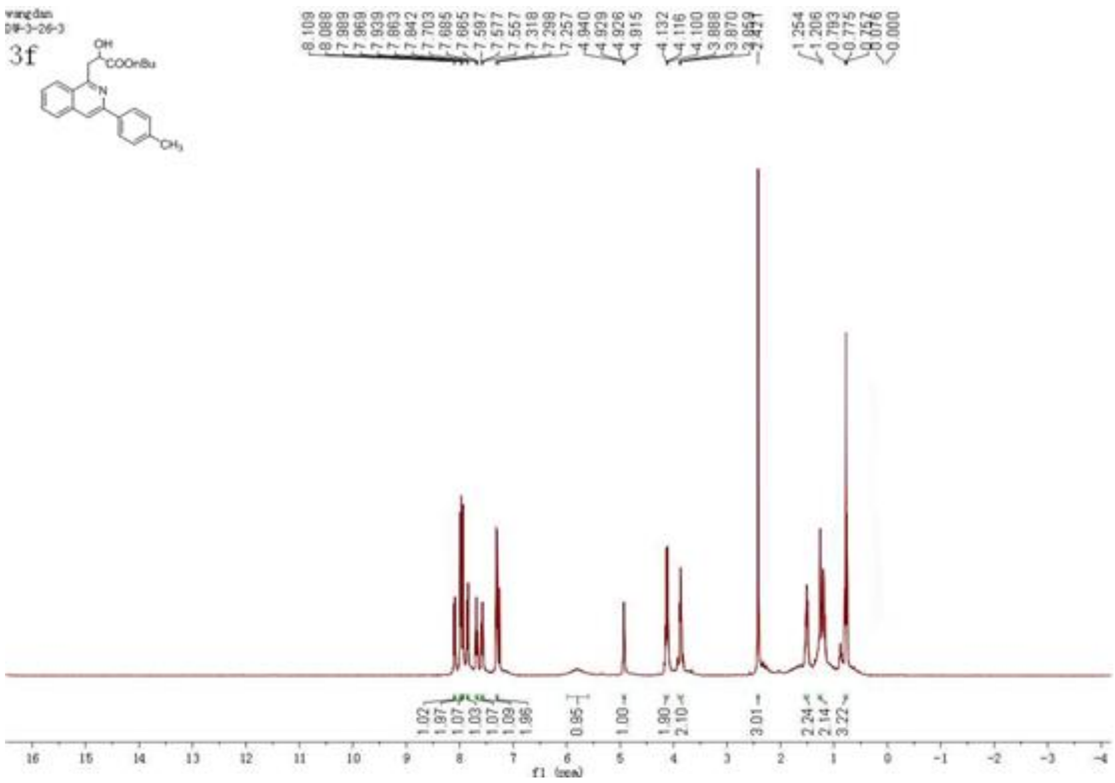
wangdan
DW-3-26-3
3f
f1 (ppm)

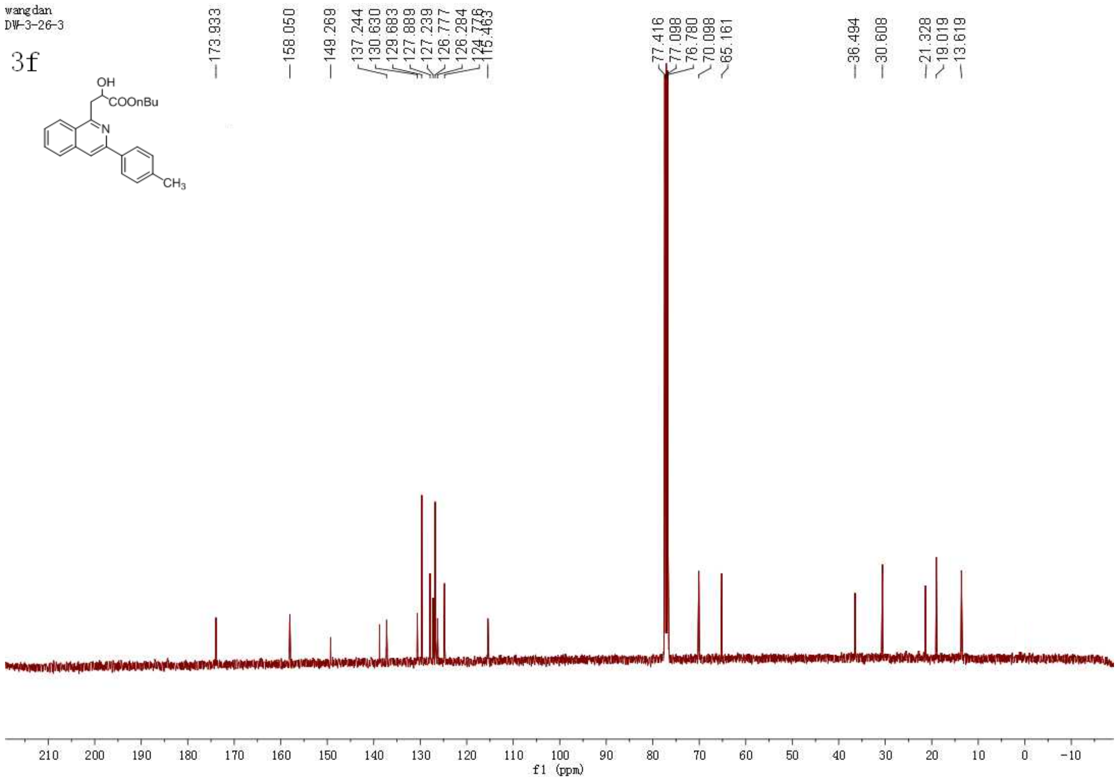
wangdan
DW-3-26-3
3f
OH
COOnBu
N
CH3
f1 (ppm)

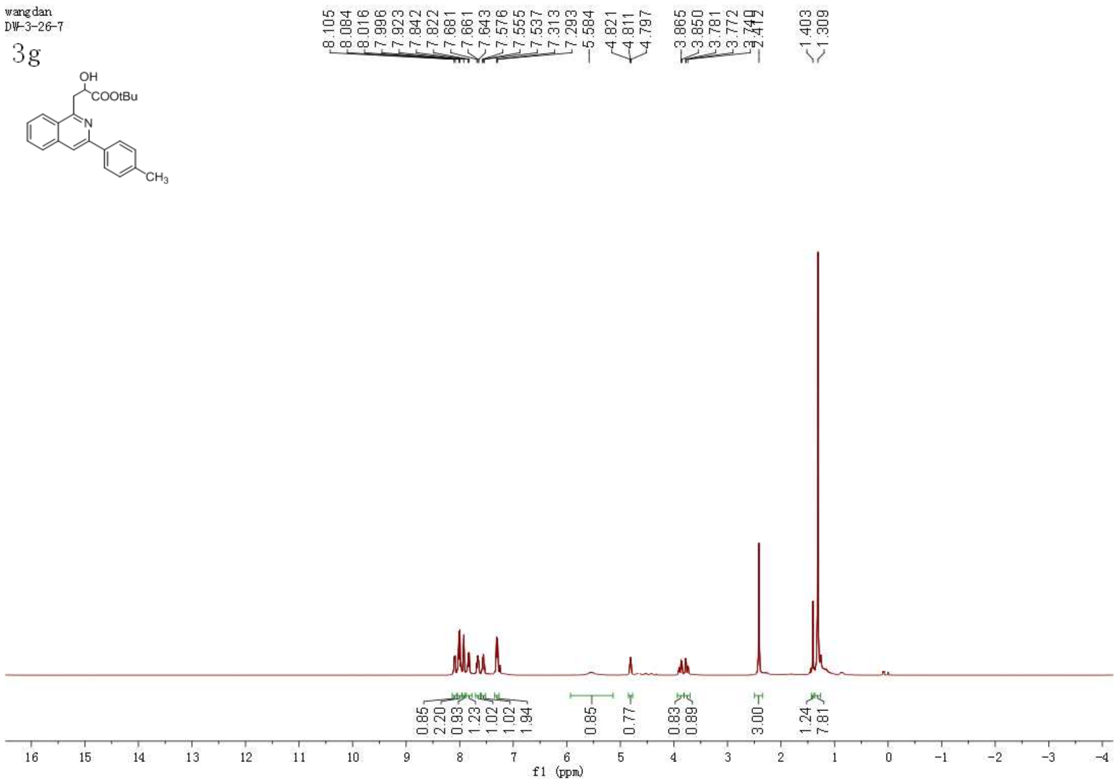
wangdan
DW-3-26-7
3g
OH
COOtBu
N
CH3
8.105 8.084 8.016 7.996 7.923 7.842 7.822 7.681 7.661 7.643 7.576 7.555 7.537 7.313 7.293 5.584 4.821 4.811 4.797 3.865 3.850 3.781 3.772 2.412 1.403 1.309
0.85 2.20 0.93 1.23 1.02 1.02 1.94 0.85 0.77 0.83 0.89 3.00 1.24 7.81
f1 (ppm)

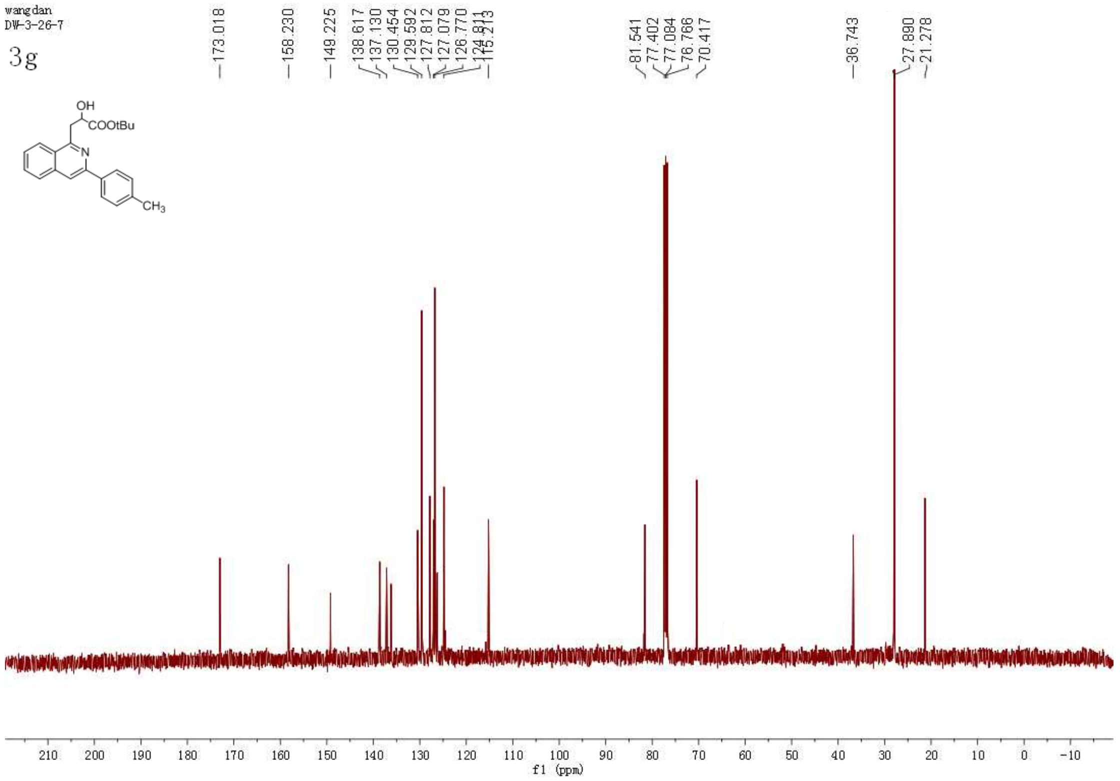
wangdan
DW-3-26-7
3g
OH
COOtBu
N
CH3
173.018 158.230 149.225 138.617 137.130 130.454 129.592 127.812 127.079 126.770 81.541 77.402 77.084 76.766 70.417 36.743 27.890 21.278
f1 (ppm)

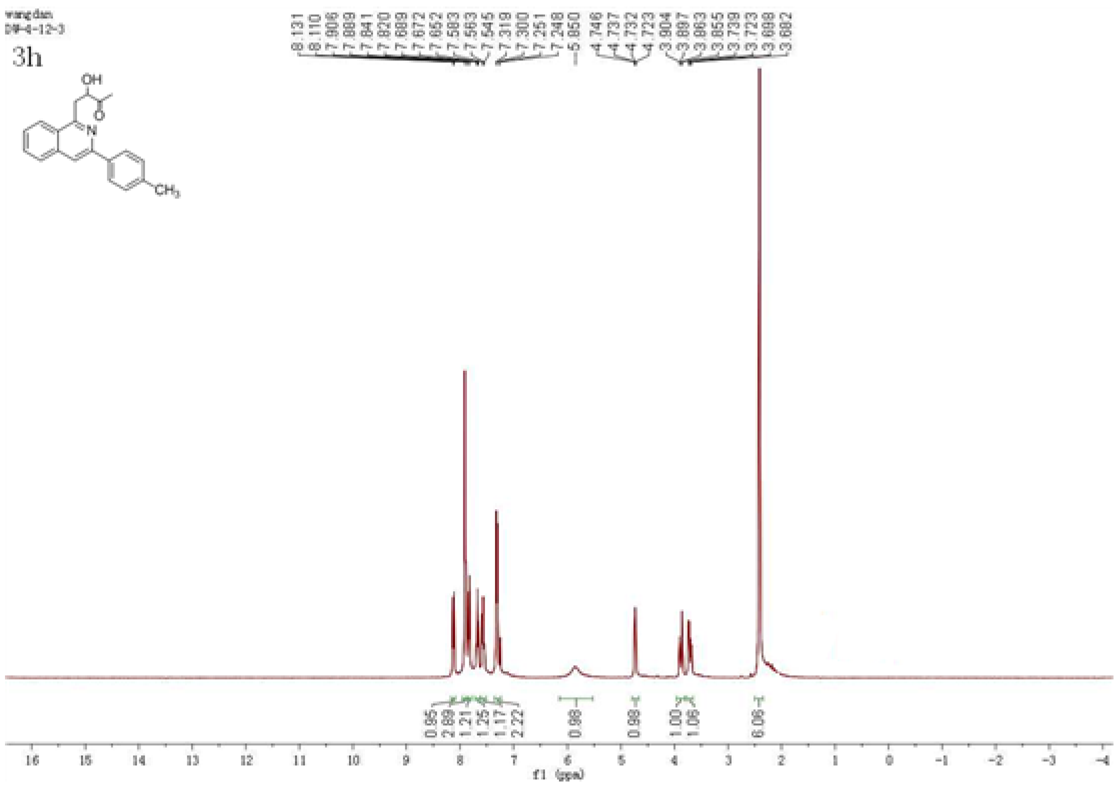
wangdan
DW-4-12-3
3h
OH
O
N
CH3
8.131 8.110 7.906 7.889 7.841 7.820 7.689 7.672 7.652 7.583 7.563 7.545 7.319 7.300 7.251 7.248 5.850 4.746 4.737 4.732 4.723 3.904 3.887 3.863 3.855 3.739 3.723 3.698 3.682
0.95 2.89 1.21 1.25 1.17 2.22 0.98 0.98 1.00 1.06 6.06
16 15 14 13 12 11 10 9 8 7 6 5 4 3 2 1 0 -1 -2 -3 -4
f1 (ppm)

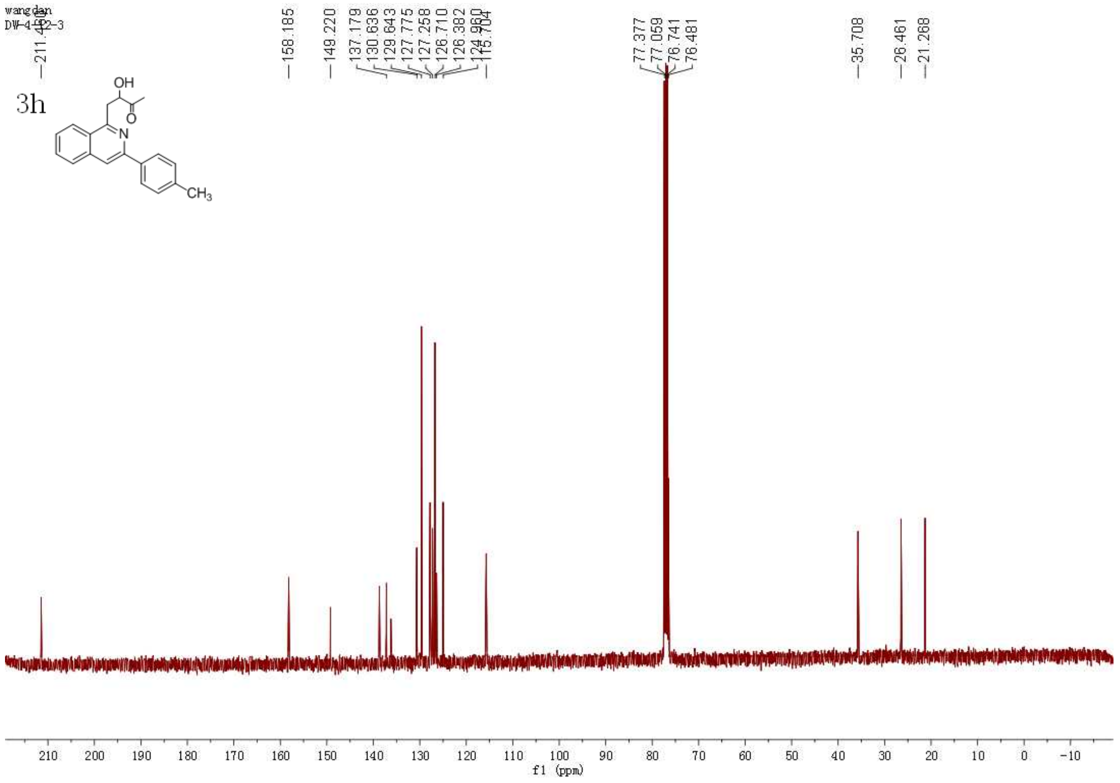
wangdan
DW-4-12-3
211.4
3h
OH
O
N
CH3
158.185 149.220 137.179 130.636 129.643 127.775 127.258 126.710 126.382 124.960 115.704
77.377 77.059 76.741 76.481
35.708 26.461 21.268
210 200 190 180 170 160 150 140 130 120 110 100 90 80 70 60 50 40 30 20 10 0 -10
f1 (ppm)

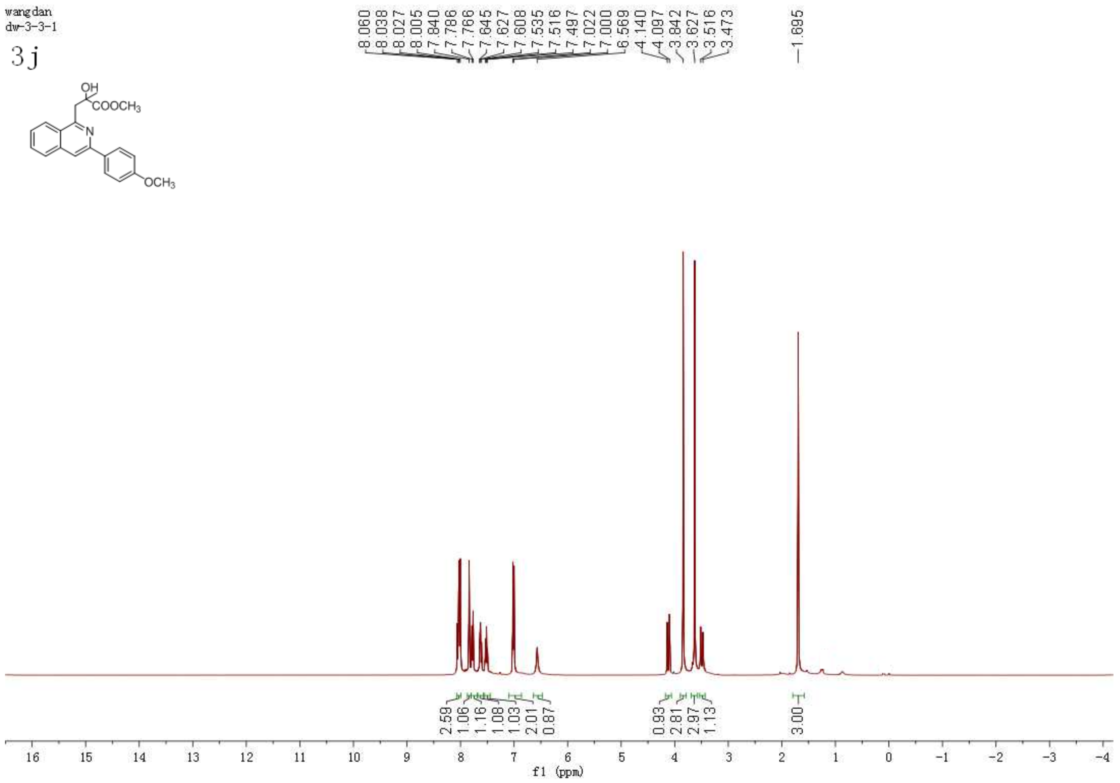
wangdan
dw-3-3-1
3j
OH
COOCH3
N
OCH3
8.060
8.038
8.027
8.005
7.840
7.786
7.766
7.645
7.627
7.608
7.535
7.516
7.497
7.022
7.000
6.569
4.140
4.097
3.842
3.627
3.516
3.473
1.695
2.59
1.06
1.16
1.08
1.03
2.01
0.87
0.93
2.81
2.97
1.13
3.00
f1 (ppm)

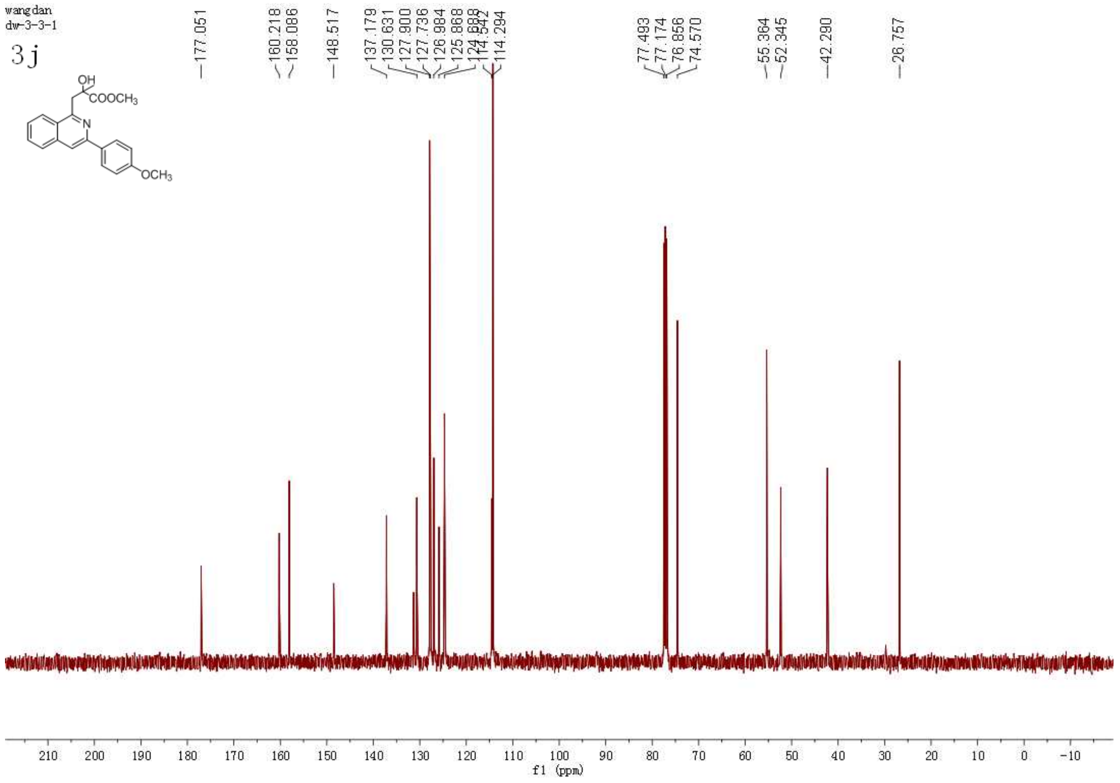
wangdan
dw-3-3-1
3j
OH
COOCH3
N
OCH3
177.051
160.218
158.086
148.517
137.179
130.631
127.900
127.736
126.984
125.868
114.542
114.294
77.493
77.174
76.856
74.570
55.364
52.345
42.290
26.757
f1 (ppm)

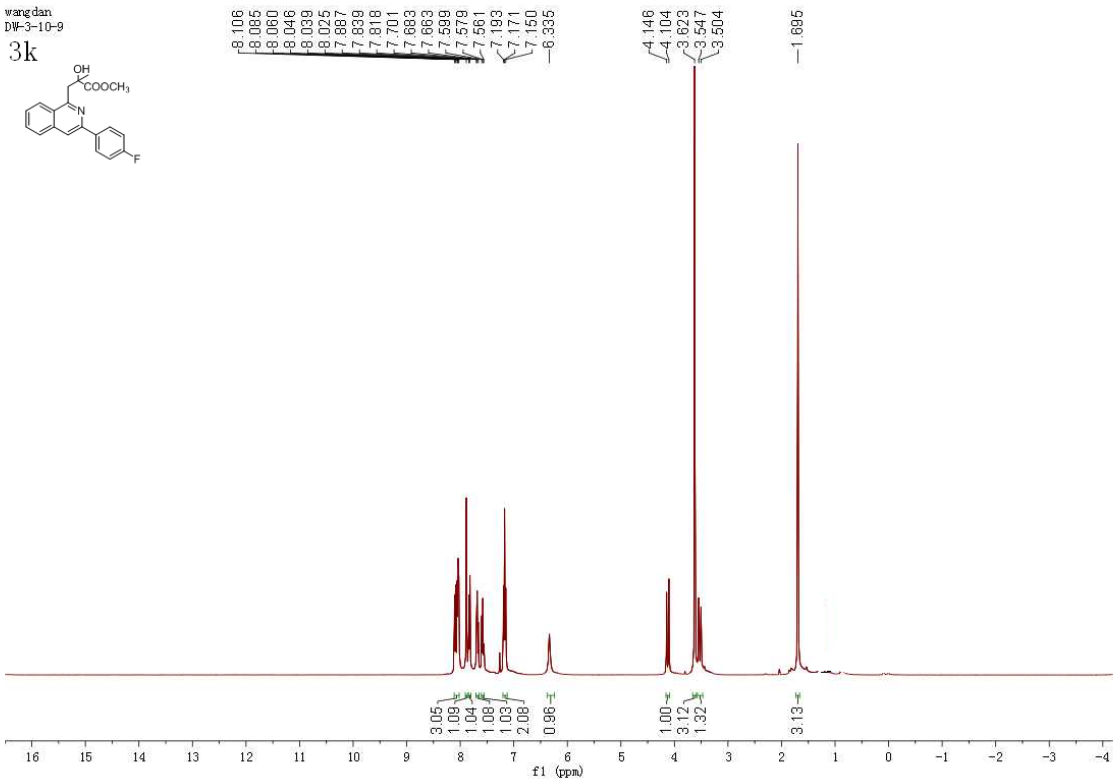

wangdan
DW-3-10-9
3k
OH
COOCH3
N
F
8.106
8.085
8.060
8.046
8.039
8.025
7.887
7.839
7.818
7.701
7.683
7.663
7.599
7.579
7.561
7.193
7.171
7.150
6.335
4.146
4.104
3.623
3.547
3.504
1.695
3.05
1.09
1.04
1.08
1.03
2.08
0.96
1.00
3.12
1.32
3.13
f1 (ppm)

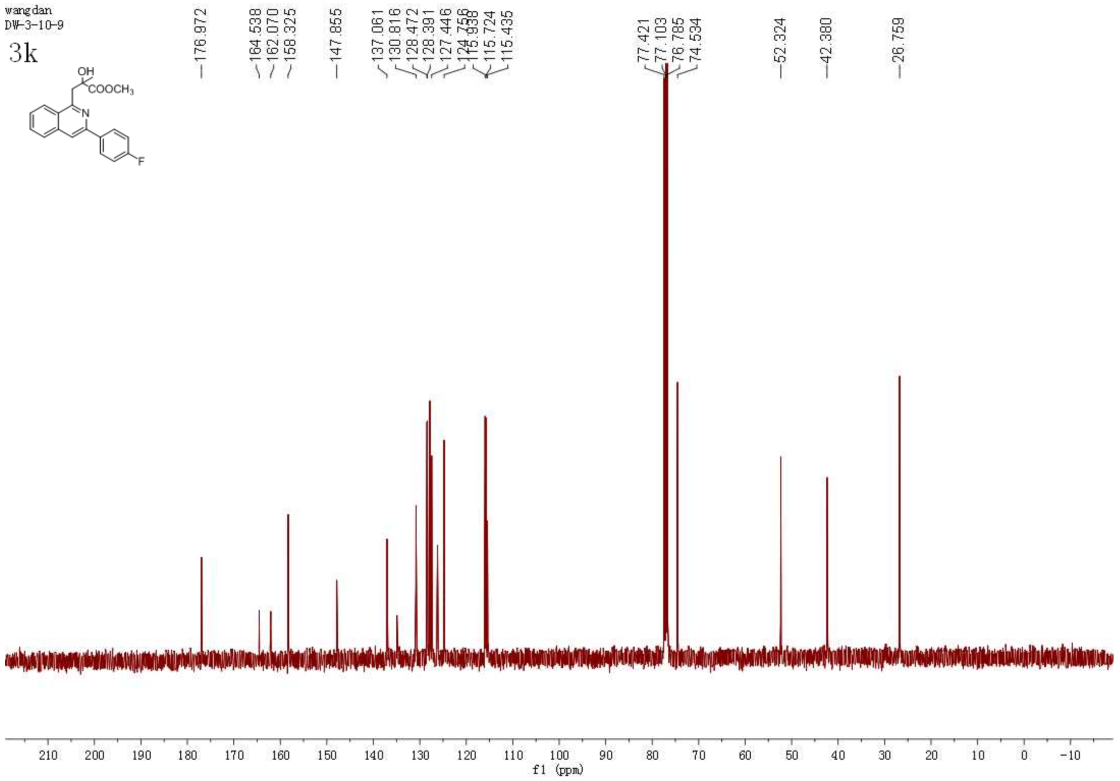

wangdan
DW-3-10-9
3k
OH
COOCH3
N
F
176.972
164.538
162.070
158.325
147.855
137.061
130.816
128.472
128.391
127.446
115.724
115.435
77.421
77.103
76.785
74.534
52.324
42.380
26.759
f1 (ppm)

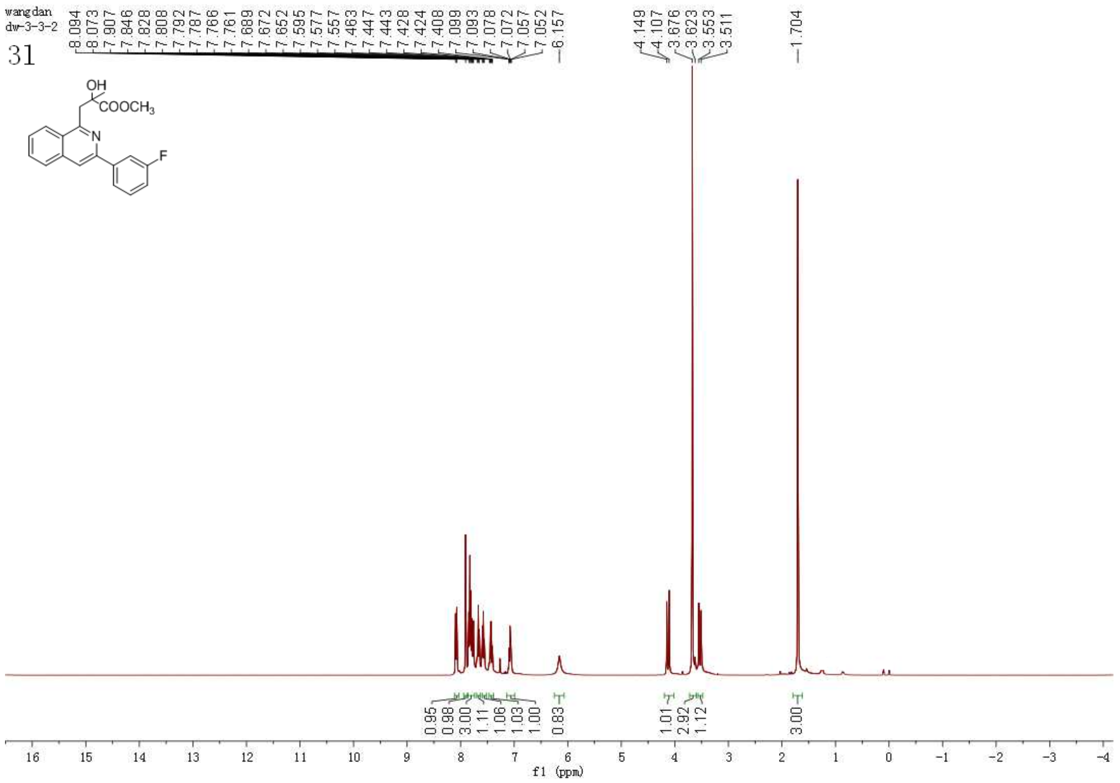
wangdan
dw-3-3-2
31
8.094
8.073
7.907
7.846
7.828
7.808
7.792
7.787
7.766
7.761
7.689
7.672
7.652
7.595
7.577
7.557
7.463
7.447
7.443
7.428
7.424
7.408
7.099
7.093
7.078
7.072
7.057
7.052
6.157
4.149
4.107
3.676
3.623
3.553
3.511
1.704
OH
COOCH3
N
F
0.95
0.98
3.00
1.11
1.06
1.03
1.00
0.83
1.01
2.92
1.12
3.00
f1 (ppm)

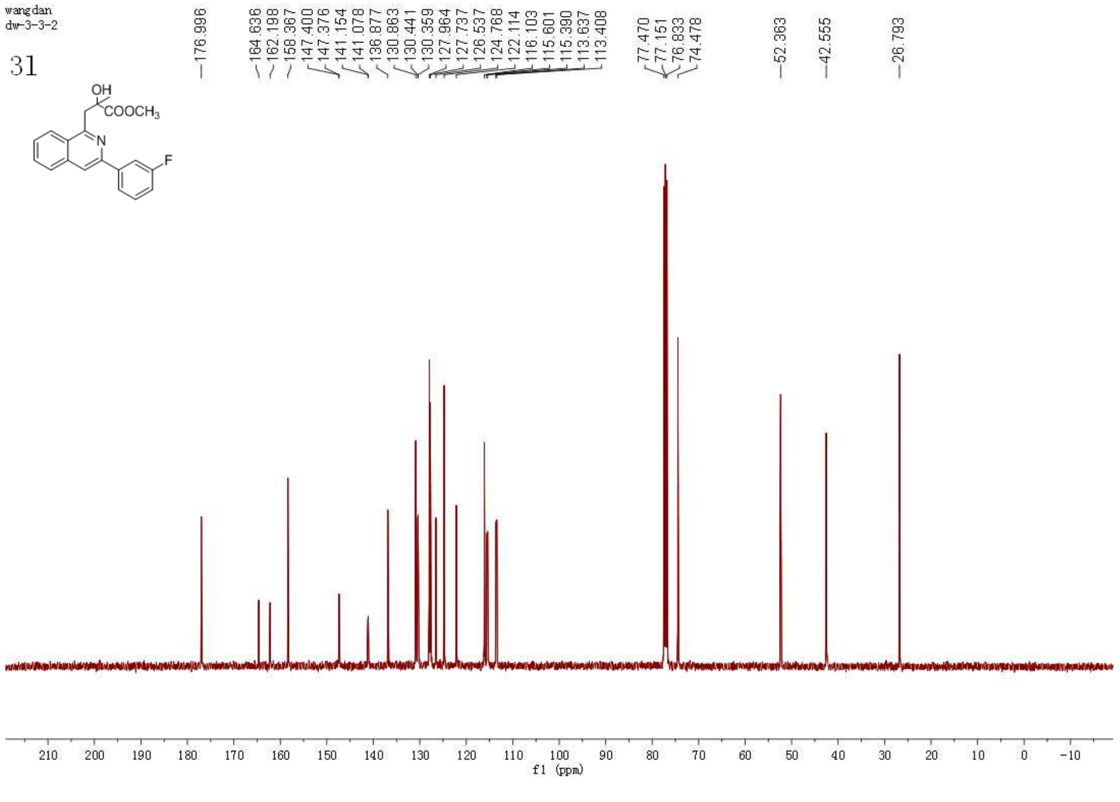
wangdan
dw-3-3-2
31
176.996
164.636
162.198
158.367
147.400
147.376
141.154
141.078
136.877
130.863
130.441
130.359
127.964
127.737
126.537
124.768
122.114
116.103
115.601
115.390
113.637
113.408
77.470
77.151
76.833
74.478
52.363
42.555
26.793
OH
COOCH3
N
F
f1 (ppm)

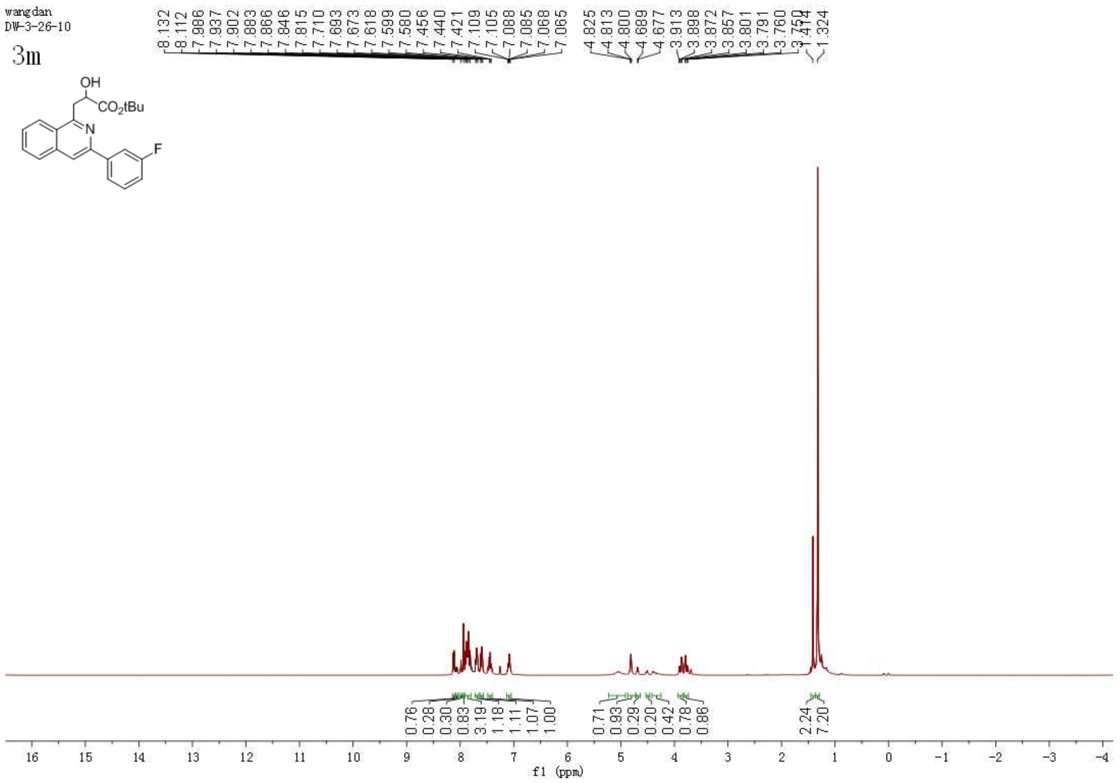
wangdan
DW-3-26-10
3m
OH
CO2tBu
N
F
8.132 8.112 7.986 7.937 7.902 7.883 7.866 7.846 7.815 7.710 7.693 7.673 7.618 7.599 7.580 7.456 7.440 7.421 7.109 7.105 7.088 7.085 7.068 7.065
4.825 4.813 4.800 4.689 4.677 3.913 3.898 3.872 3.857 3.801 3.791 3.760 3.750 1.414 1.324
0.76 0.28 0.30 0.83 3.19 1.18 1.11 1.07 1.00
0.71 0.93 0.29 0.20 0.42 0.78 0.86
2.24 7.20
f1 (ppm)

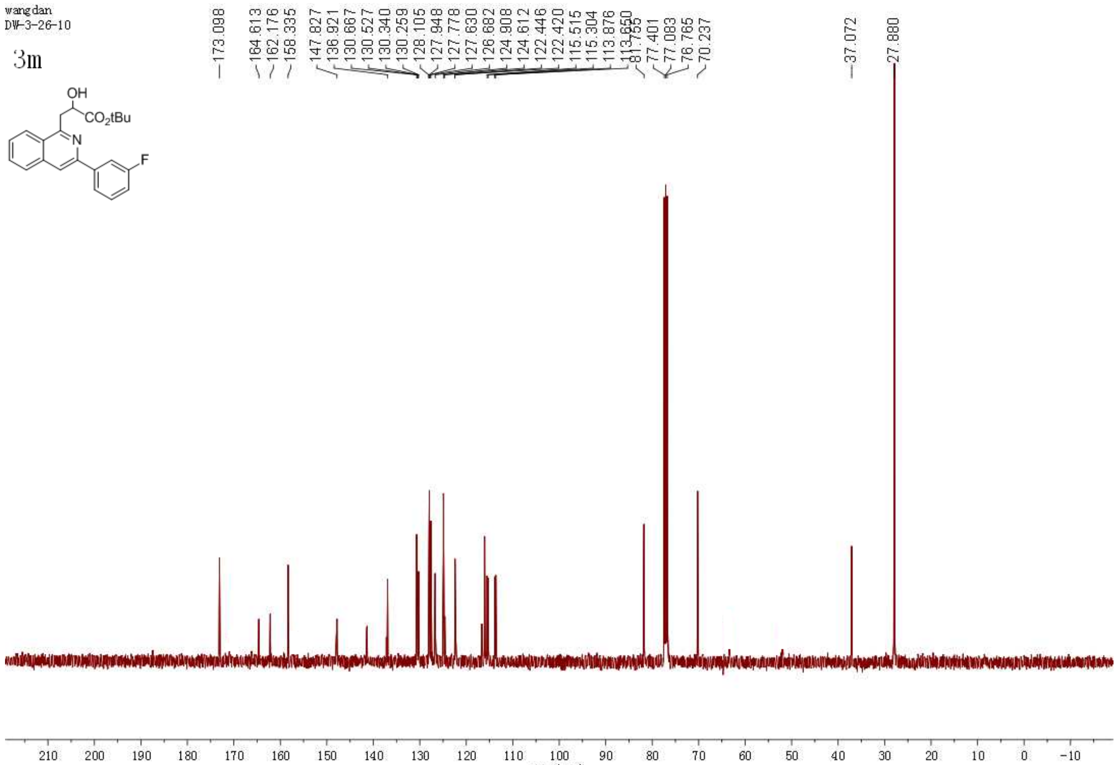
wangdan
DW-3-26-10
3m
OH
CO2tBu
N
F
173.098
164.613 162.176 158.335
147.827 136.921 130.667 130.527 130.340 130.259 128.105 127.948 127.778 127.630 126.682 124.908 124.612 122.446 122.420 115.515 115.304 113.876 113.650
81.755 77.401 77.083 76.765 70.237
37.072
27.880
f1 (ppm)

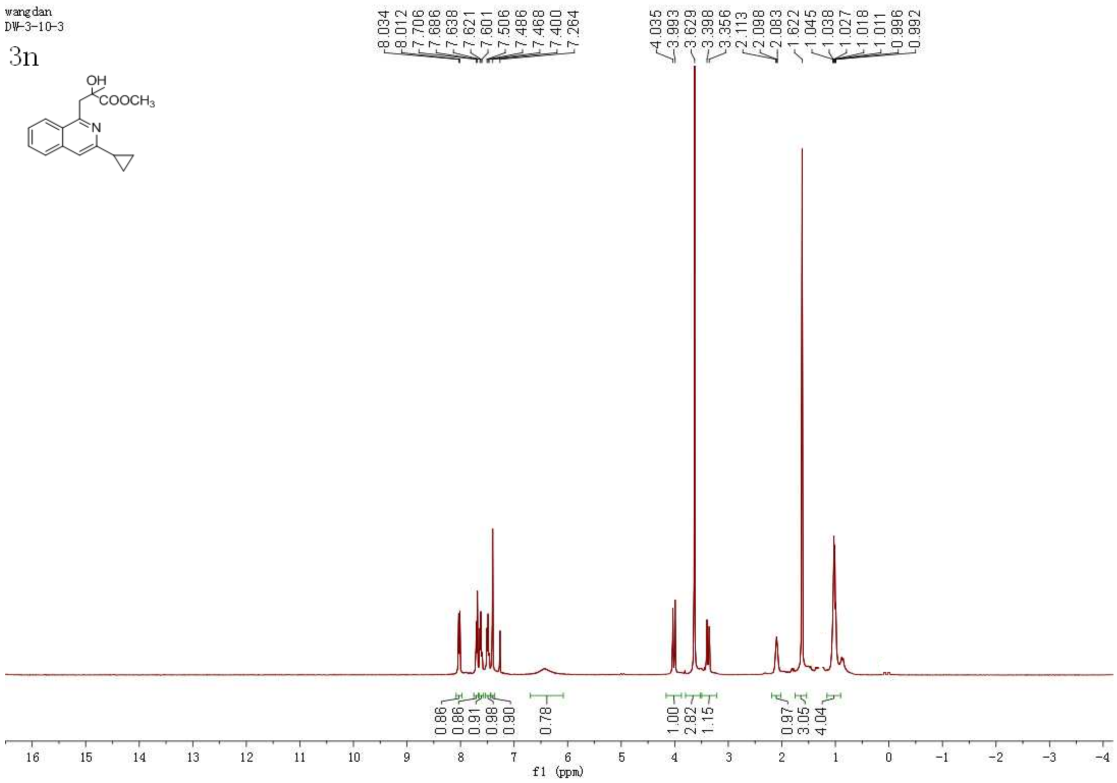
wangdan
DW-3-10-3
3n
OH
COOCH3
N
8.034
8.012
7.706
7.686
7.638
7.621
7.601
7.506
7.486
7.468
7.400
7.264
4.035
3.993
3.629
3.398
3.356
2.113
2.098
2.083
1.622
1.045
1.038
1.027
1.018
1.011
0.996
0.992
0.86
0.86
0.91
0.98
0.90
0.78
1.00
2.82
1.15
0.97
3.05
4.04
f1 (ppm)

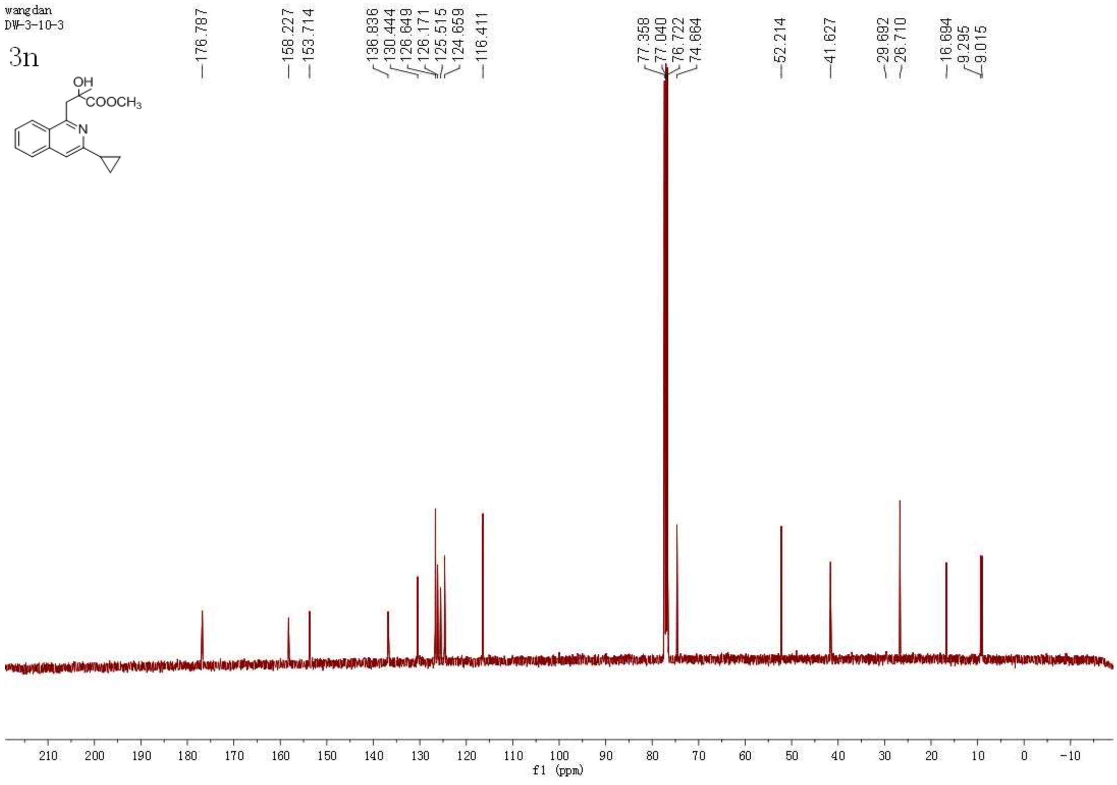
wangdan
DW-3-10-3
3n
OH
COOCH3
N
176.787
158.227
153.714
136.836
130.444
126.649
126.171
125.515
124.659
116.411
77.358
77.040
76.722
74.664
52.214
41.627
29.692
26.710
16.694
9.295
9.015
f1 (ppm)

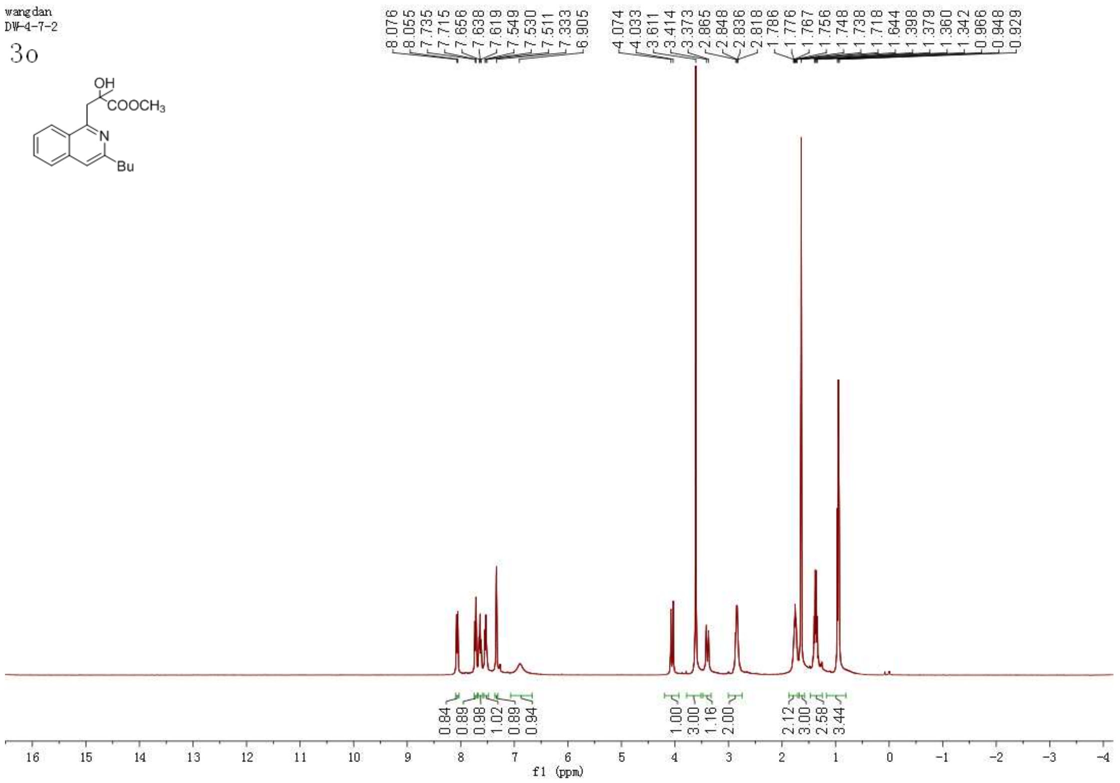
wangdan
DW-4-7-2
3o
OH
COOCH3
N
Bu
8.076 8.055 7.735 7.715 7.656 7.638 7.619 7.549 7.530 7.511 7.333 6.905
4.074 4.033 3.611 3.414 3.373 2.865 2.848 2.836 2.818 1.786 1.776 1.767 1.756 1.748 1.738 1.718 1.644 1.398 1.379 1.360 1.342 0.966 0.948 0.929
0.84 0.89 0.98 1.02 0.89 0.94
1.00 3.00 1.16 2.00
2.12 3.00 2.58 3.44
16 15 14 13 12 11 10 9 8 7 6 5 4 3 2 1 0 −1 −2 −3 −4
f1 (ppm)

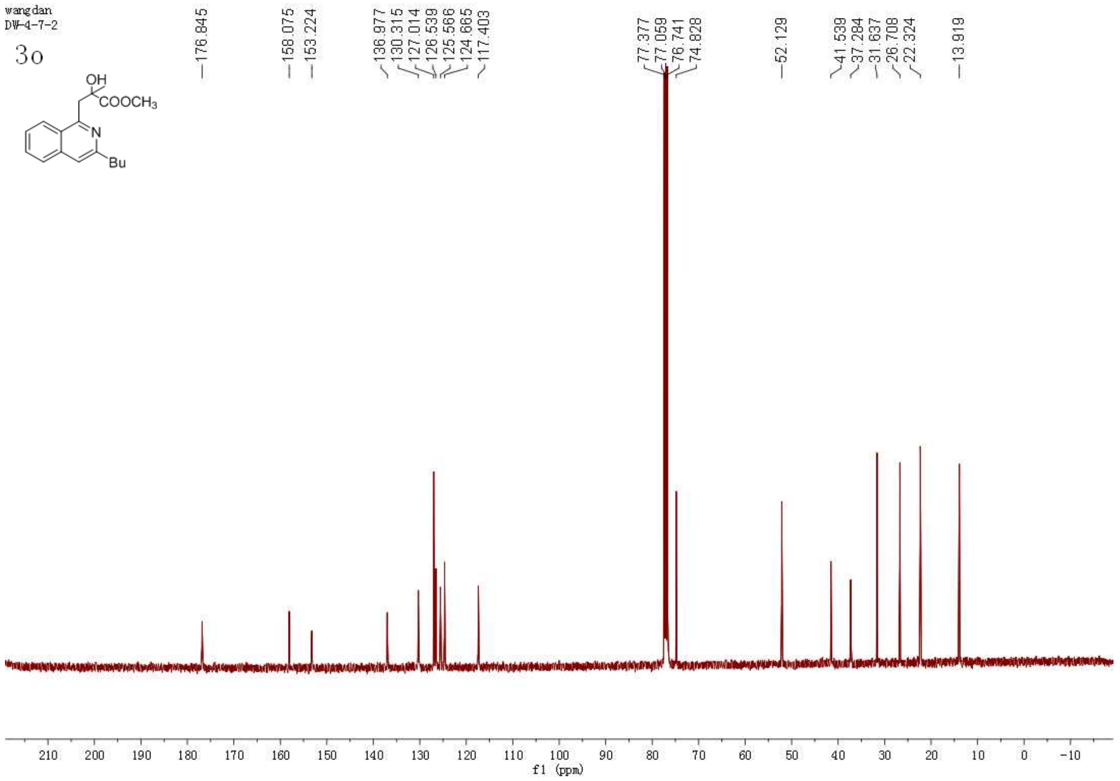
wangdan
DW-4-7-2
3o
OH
COOCH3
N
Bu
176.845
158.075 153.224
136.977 130.315 127.014 126.539 125.566 124.665 117.403
77.377 77.059 76.741 74.828
52.129
41.539 37.284 31.637 26.708 22.324
13.919
210 200 190 180 170 160 150 140 130 120 110 100 90 80 70 60 50 40 30 20 10 0 −10
f1 (ppm)

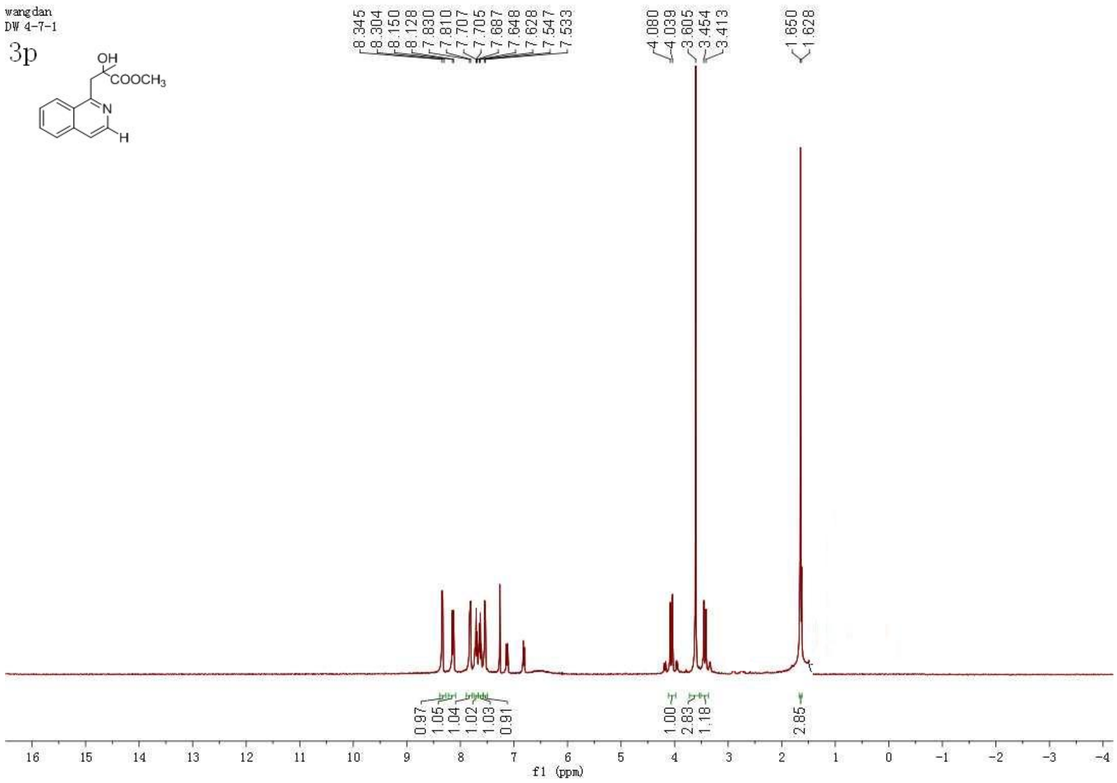
wangdan
DW 4-7-1
3p
OH
COOCH3
N
H
8.345
8.304
8.150
8.128
7.830
7.810
7.707
7.705
7.687
7.648
7.628
7.547
7.533
4.080
4.039
3.605
3.454
3.413
1.650
1.628
0.97
1.05
1.04
1.02
1.03
0.91
1.00
2.83
1.18
2.85
f1 (ppm)

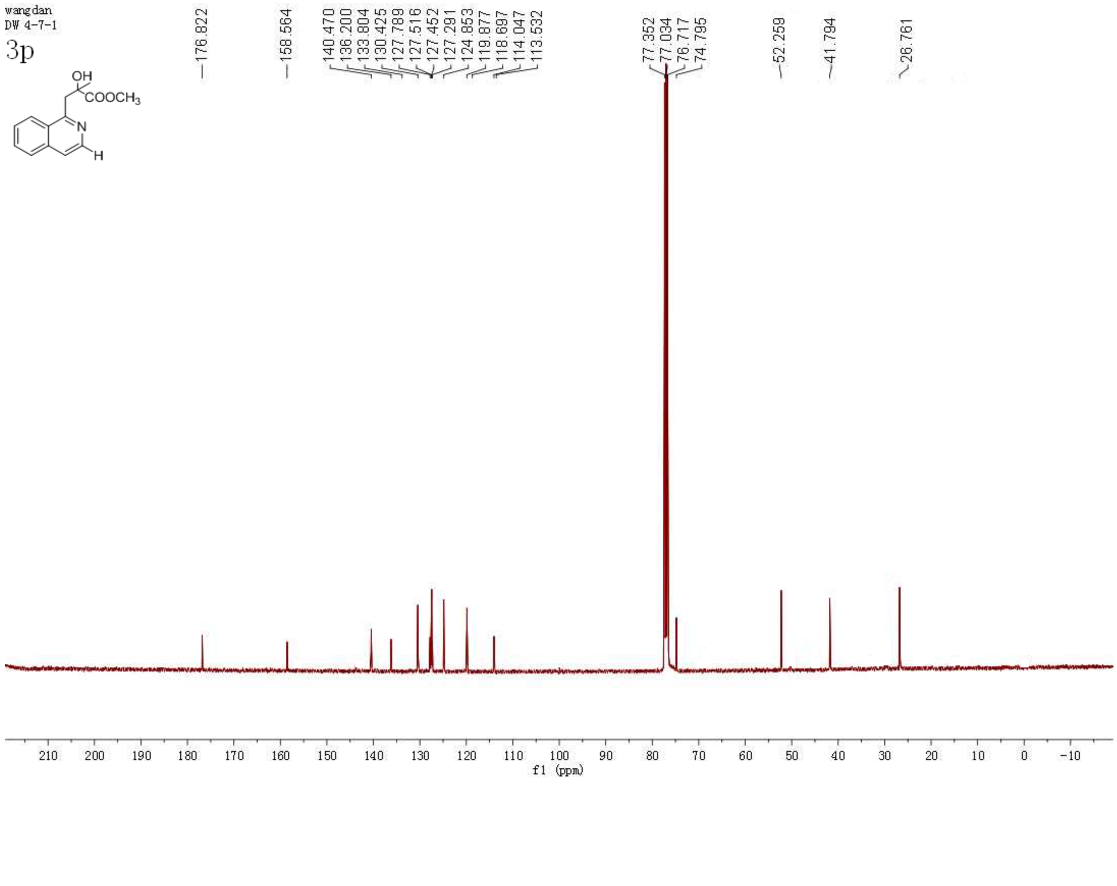
wangdan
DW 4-7-1
3p
OH
COOCH3
N
H
176.822
158.564
140.470
136.200
133.804
130.425
127.789
127.516
127.452
127.291
124.853
119.877
118.697
114.047
113.532
77.352
77.034
76.717
74.795
52.259
41.794
26.761
f1 (ppm)

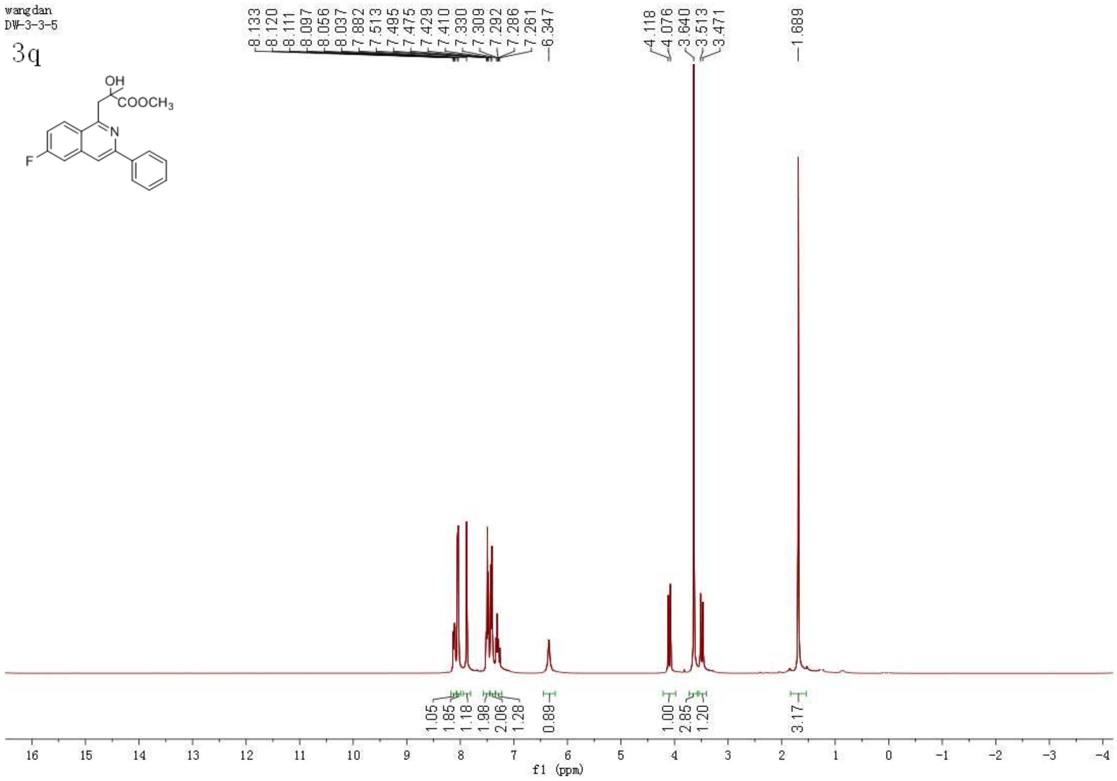
wangdan
DW-3-3-5
3q
OH
COOCH3
N
F
8.133
8.120
8.111
8.097
8.056
8.037
7.882
7.513
7.495
7.475
7.429
7.410
7.330
7.309
7.292
7.286
7.261
6.347
4.118
4.076
3.640
3.513
3.471
1.689
1.05
1.85
1.18
1.98
2.06
1.28
0.89
1.00
2.85
1.20
3.17
f1 (ppm)

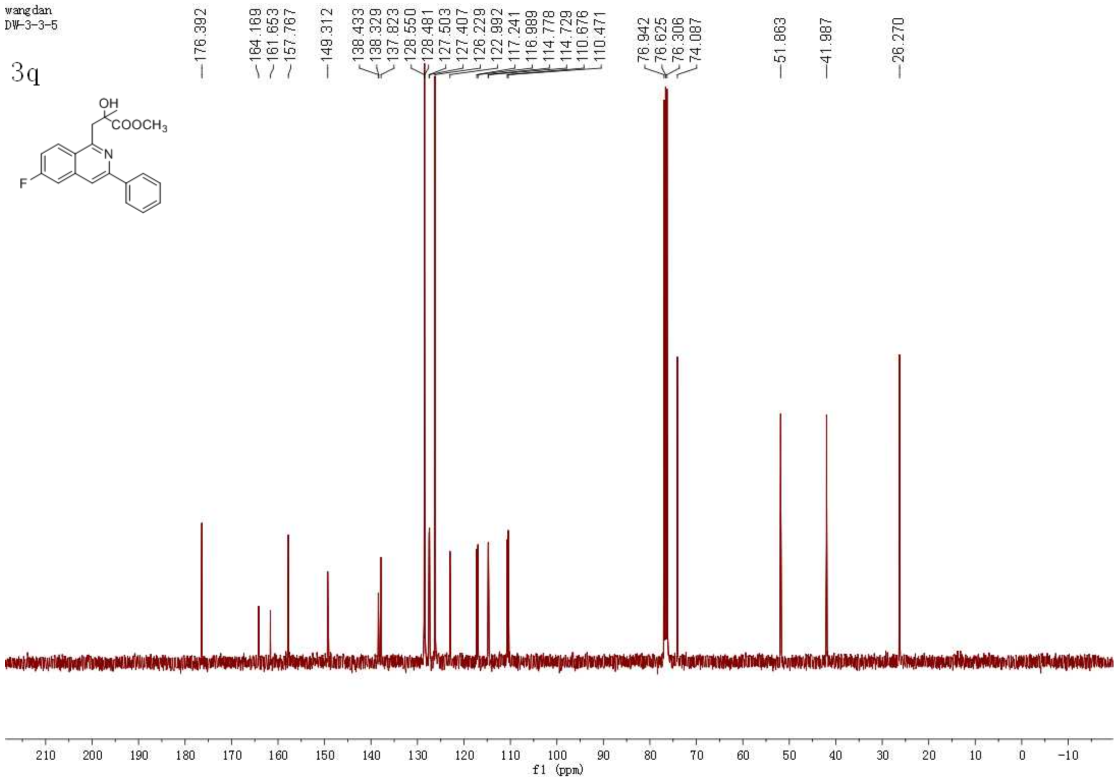
wangdan
DW-3-3-5
3q
OH
COOCH3
N
F
176.392
164.169
161.653
157.767
149.312
138.433
138.329
137.823
128.550
128.481
127.503
127.407
126.229
122.992
117.241
116.989
114.778
114.729
110.676
110.471
76.942
76.625
76.306
74.087
51.863
41.987
26.270
f1 (ppm)

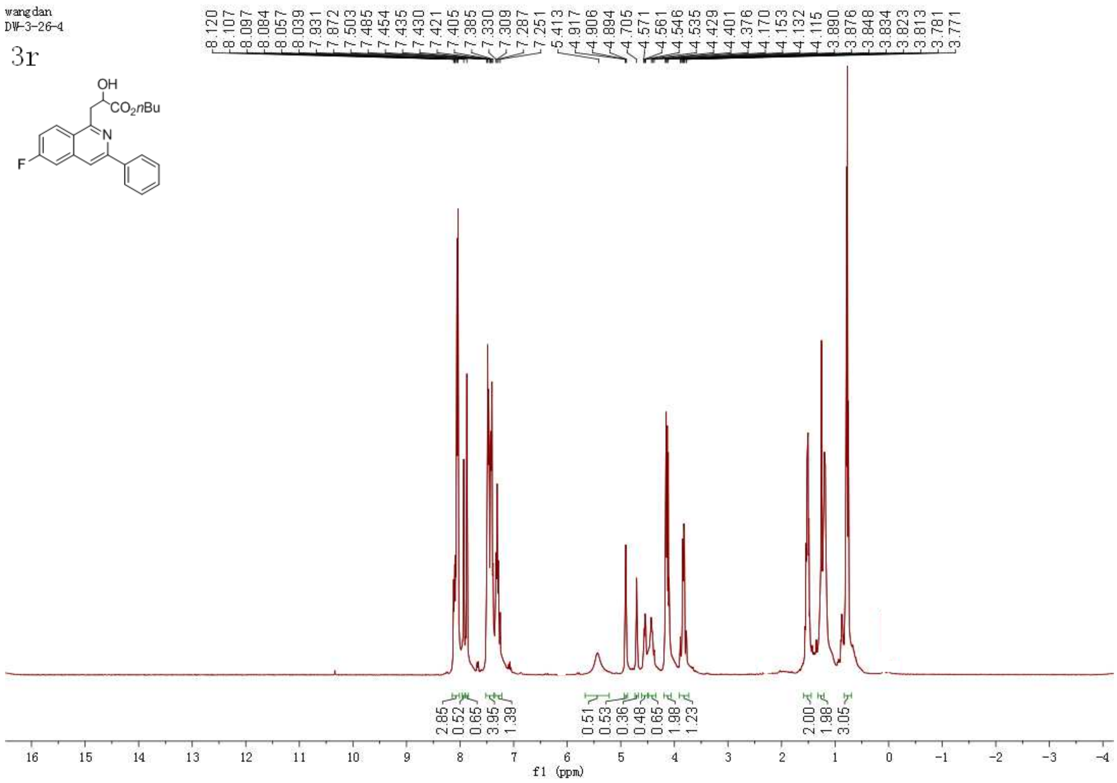
wangdan
DW-3-26-4
3r
OH
CO2nBu
N
F
8.120
8.107
8.097
8.084
8.057
8.039
7.931
7.872
7.503
7.485
7.454
7.435
7.430
7.421
7.405
7.385
7.330
7.309
7.287
7.251
5.413
4.917
4.906
4.894
4.705
4.571
4.561
4.546
4.535
4.429
4.401
4.376
4.170
4.153
4.132
4.115
3.890
3.876
3.848
3.834
3.823
3.813
3.781
3.771
2.85
0.52
0.65
3.95
1.39
0.51
0.53
0.36
0.48
0.65
1.98
1.23
2.00
1.98
3.05
16 15 14 13 12 11 10 9 8 7 6 5 4 3 2 1 0 −1 −2 −3 −4
f1 (ppm)

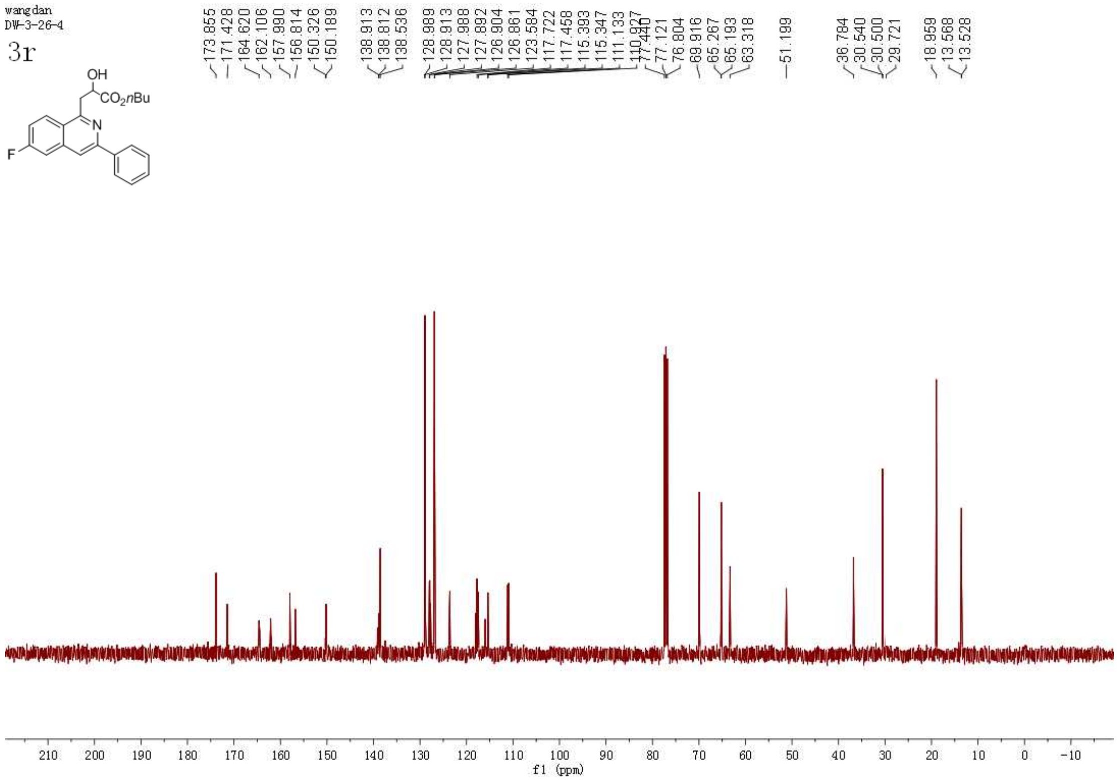
wangdan
DW-3-26-4
3r
OH
CO2nBu
N
F
173.855
171.428
164.620
162.106
157.990
156.814
150.326
150.189
138.913
138.812
138.536
128.989
128.913
127.988
127.892
126.904
126.861
123.584
117.722
117.458
115.393
115.347
111.133
110.927
77.440
77.121
76.804
69.916
65.267
65.193
63.318
51.199
36.784
30.540
30.500
29.721
18.959
13.568
13.528
210 200 190 180 170 160 150 140 130 120 110 100 90 80 70 60 50 40 30 20 10 0 −10
f1 (ppm)

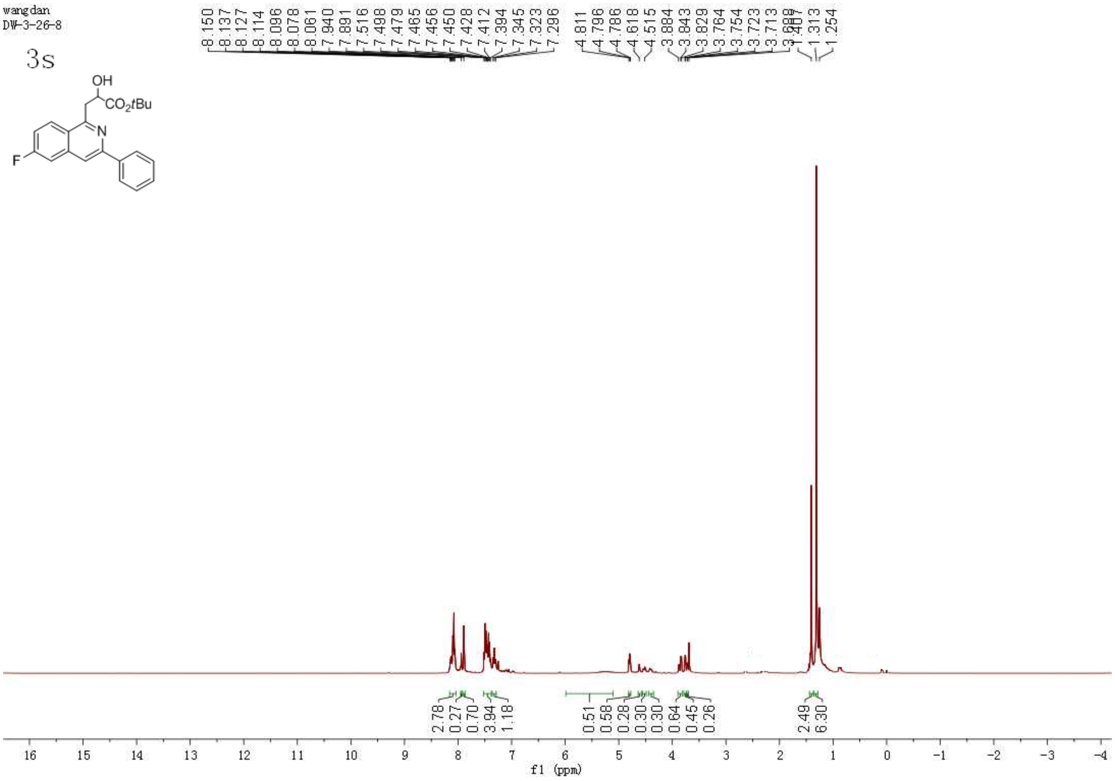
wangdan
DW-3-26-8
3s
OH
CO2tBu
N
F
8.150 8.137 8.127 8.114 8.096 8.078 8.061 7.940 7.891 7.516 7.498 7.479 7.465 7.456 7.450 7.428 7.412 7.394 7.345 7.323 7.296
4.811 4.796 4.786 4.618 4.515 3.884 3.843 3.829 3.764 3.754 3.723 3.713
1.313 1.254
2.78 0.27 0.70 3.94 1.18
0.51 0.58 0.28 0.30 0.30 0.64 0.45 0.26
2.49 6.30
f1 (ppm)

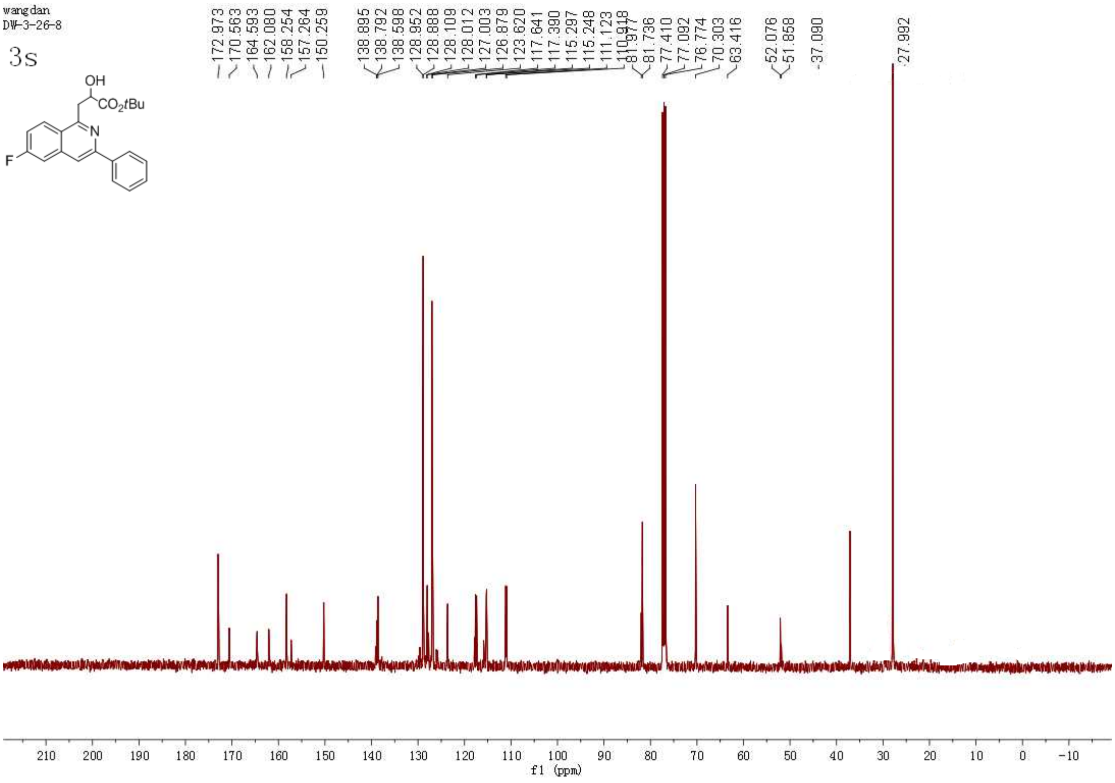
wangdan
DW-3-26-8
3s
OH
CO2tBu
N
F
172.973 170.563 164.593 162.080 158.254 157.264 150.259
138.895 138.792 138.598 128.952 128.888 128.109 128.012 127.003 126.879 123.620 117.641 117.390 115.297 115.248 111.123
81.736 77.410 77.092 76.774 70.303 63.416
52.076 51.858
37.090
27.992
f1 (ppm)

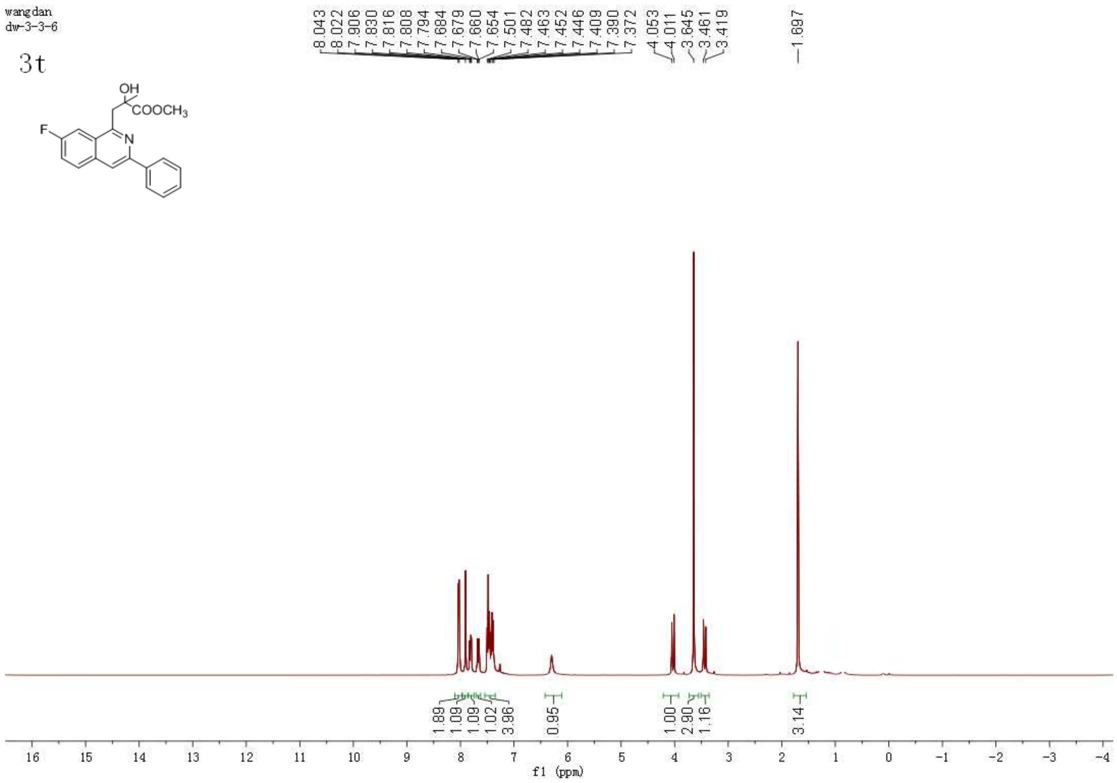

wangdan
dw-3-3-6
3t
OH
COOCH3
F
N
8.043
8.022
7.906
7.830
7.816
7.808
7.794
7.684
7.679
7.660
7.654
7.501
7.482
7.463
7.452
7.446
7.409
7.390
7.372
4.053
4.011
3.645
3.461
3.419
1.697
1.89
1.09
1.09
1.02
3.96
0.95
1.00
2.90
1.16
3.14
f1 (ppm)

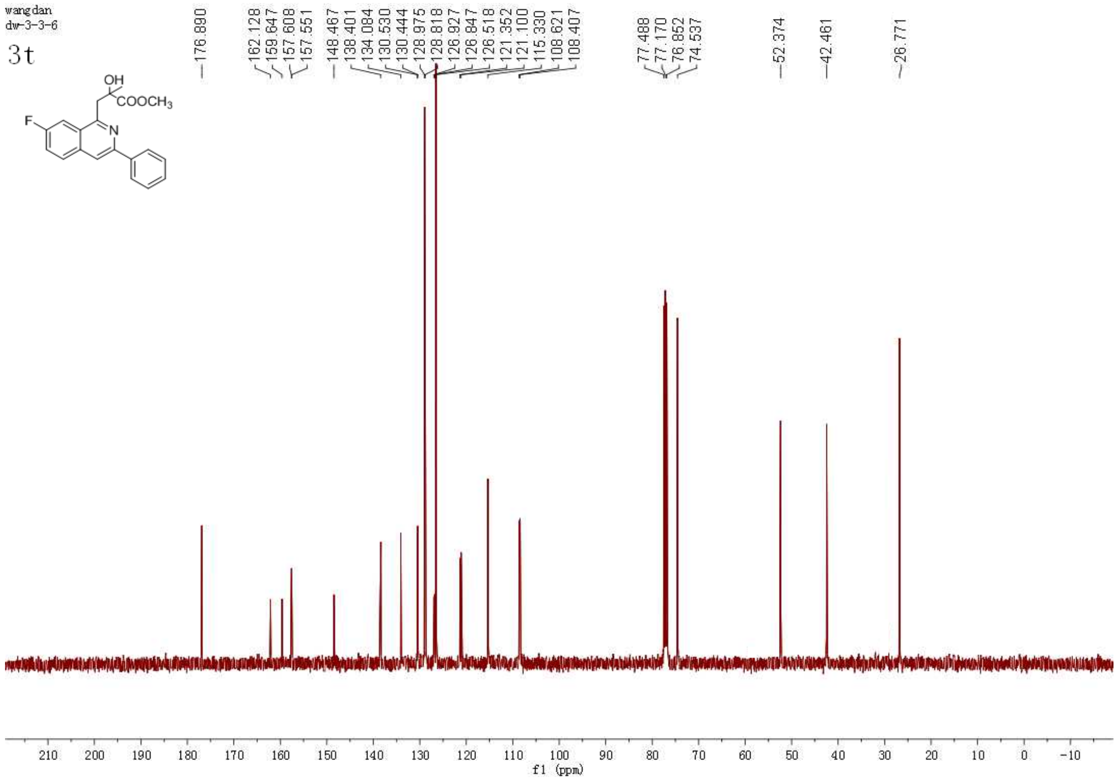

wangdan
dw-3-3-6
3t
OH
COOCH3
F
N
176.890
162.128
159.647
157.608
157.551
148.467
138.401
134.084
130.530
130.444
128.975
128.818
126.927
126.847
126.518
121.352
121.100
115.330
108.621
108.407
77.488
77.170
76.852
74.537
52.374
42.461
26.771
f1 (ppm)

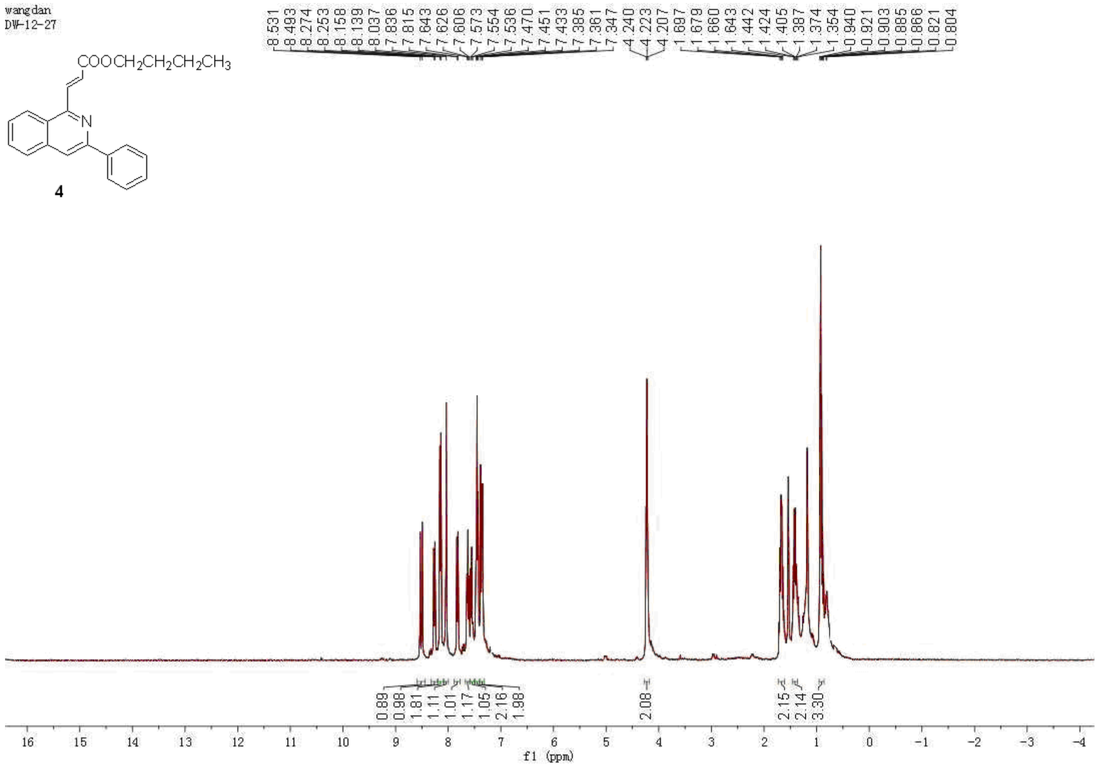
wangdan
DW-12-27
COOCH2CH2CH2CH3
N
4
f1 (ppm)

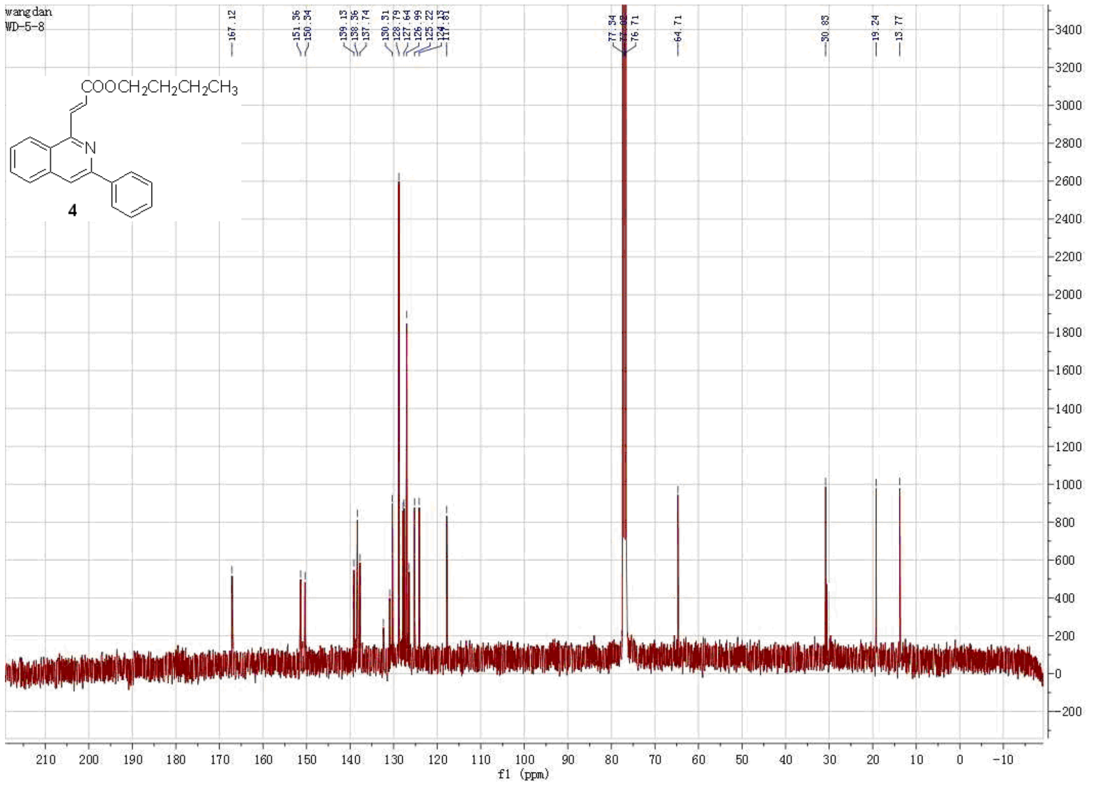
wangdan
WD-5-8
COOCH2CH2CH2CH3
N
4
f1 (ppm)
